# The Invasive Anemone *Condylactis* sp. of the Coral Reef as a Source of Sulfur- and Nitrogen-Containing Metabolites and Cytotoxic 5,8-Epidioxy Steroids

**DOI:** 10.3390/metabo13030392

**Published:** 2023-03-07

**Authors:** Atallah F. Ahmed, Chang-Feng Dai, Yao-Haur Kuo, Jyh-Horng Sheu

**Affiliations:** 1 Department of Marine Resources, National Sun Yat-sen University, Kaohsiung 804, Taiwan; 2 Department of Pharmacognosy, College of Pharmacy, King Saud University, Riyadh 11451, Saudi Arabia; 3 Department of Pharmacognosy, Faculty of Pharmacy, Mansoura University, Mansoura 35516, Egypt; 4 Institute of Oceanography, National Taiwan University, Taipei 106, Taiwan; 5 Division of Herbal Drugs and Natural Products, National Research Institute of Chinese Medicine, Taipei 112, Taiwan; 6 Institute of Natural Products, Kaohsiung Medical University, Kaohsiung 807, Taiwan; 7 Department of Medical Research, China Medical University Hospital, China Medical University, Taichung 404, Taiwan; 8 Frontier Center for Ocean Science and Technology, National Sun Yat-sen University, Kaohsiung 804, Taiwan; 9 Doctoral Degree Program in Marine Biotechnology, National Sun Yat-sen University, Kaohsiung 804, Taiwan

**Keywords:** anemone, *Condylactis* sp., marine alkaloids, thioniacinamide, natural 1,2,4-thiadiazole, cytotoxicity, 5,8-epidioxysteroids, ecological impact

## Abstract

The *Condylactis*-genus anemones were examined for their proteinaceous poisons over 50 years ago. On the other hand, the current research focuses on isolating and describing the non-proteinaceous secondary metabolites from the invasive *Condylactis* anemones, which help take advantage of their population outbreak as a new source of chemical candidates and potential drug leads. From an organic extract of *Condylactis* sp., a 1,2,4-thiadiazole-based alkaloid, identified as 3,5-bis(3-pyridinyl)-1,2,4-thiadiazole (**1**), was found to be a new natural alkaloid despite being previously synthesized. The full assignment of NMR data of compound **1**, based on the analysis of 2D NMR correlations, is reported herein for the first time. The proposed biosynthetic precursor thionicotinamide (**2**) was also isolated for the first time from nature along with nicotinamide (**3**), uridine (**5**), hypoxanthine (**6**), and four 5,8-epidioxysteroids (**7**–**10**). A major secondary metabolite (−)-betonicine (**4**) was isolated from *Condylactis* sp. and found for the first time in marine invertebrates. The four 5,8-epidioxysteroids, among other metabolites, exhibited cytotoxicity (IC_50_ 3.5–9.0 μg/mL) toward five cancer cell lines.

## 1. Introduction

Marine organisms have been well-recognized as a rich source of novel metabolites with interesting biological activities [1]. Cnidaria, including sea anemones (family Actiniidae), is known to produce a variety of venoms from which many proteinaceous (peptides and enzymes) [2,3,4,5,6,7,8,9,10,11] and non-proteinaceous (biogenic amines, quaternary ammonium compounds, and purines) constituents [12] have been identified. The anemone venom toxins have ecological and biotechnological importance [12,13]. They help sea anemones catch prey, defend against predators, and avoid competitors [4]. Sodium and potassium channel modulating [2,5,6,14,15], pore-forming [3,7], lethal cytolytic [8], cardio stimulatory [9], hemolytic [9,10], and arrhythmogenic [11] proteins have been discovered in some sea anemones, including *Condylactis species* [11,14,15]. However, some marine fungi isolated from sea anemones were found to be responsible for producing certain antibacterial and cytotoxic metabolites, such as anthraquinone derivatives [16]. Anemones of the genus *Condylactis* are widely found in the Red Sea, the Mediterranean Sea, and the Caribbean Sea [17,18]. Since more than 50 years ago, researchers have examined *Condylactis*-genus anemones for their proteinaceous poisons [11,19], enzymes including basic phospholipase A(2) [20], and nucleic acids [14]. The specimen of the current study was collected in 2003 during the population outbreak of the sea anemones of *Condylactis*, which induced a local phase shift of the coral reef from the *Acropora*-dominant to the *Condylactis*-dominant community [21]. The occupation of the coral reef by anemones of the *Condylactis* genus may subsequently reduce the recruitment of coral larvae, thus damaging the coral community’s recovery. The causes of this *Condylactis* outbreak are not clearly known but were considered to be relevant to the consequences of Typhoon Herb (Typhoon Huaning) in 1996, the mass coral bleaching in 1997, landslides, sewage run-off, and tourism-related ecological impact in the Kenting National Park, Taiwan [22]. Another outbreak of *Condylactis nanwanensis* in 2004 was noticed in Nanwan Bay, Southern Taiwan, which is anticipated to be an invasive Caribbean species introduced via the aquarium trade. Since it kills coral tissue and weakens coral skeletons, this anemone has severely harmed coral reefs [23]. The invasive behavior of corallimorph sea anemone *Condylactis* was also recorded in the coral reefs of the Lakshadweep Archipelago, India [24]. Coral reefs are important for the economy because they provide millions of jobs and contribute to fishing, tourism, and medicine. However, coral reefs are threatened by many factors, including invasive anemones that can overgrow and smother corals. To slow the current rate of coral degradation, all of the countries of the affected regions have or are preparing monitoring and management plans for coral reefs as well as marine protected areas (MPAs) development [23]. Large anemone harvesting not only benefits the reef’s marine ecosystem, but following careful chemical investigation, it may also turn this hazardous organism into valuable sources of biologically active compounds. The absence of reports dealing with the organic soluble secondary metabolites, except for 6-bromo-5,9-eicosadienoic acid [25], prompted us to chemically and biologically investigate the non-proteinaceous metabolites of *Condylactis* sp. growing in the Southern Taiwanese water. The aim was to recruit new potential marine resources useful for future biomedical applications and to inspire ecological stability research. The chemical investigation was carried out mainly on one of the MeOH-soluble fractions of the organism’s crude extract, which showed an in vitro cytotoxicity (IC_50_ ~ 18.0 μg/mL) against two human cancer cell lines.

## 2. Materials and Methods

### 2.1. General Procedures

Optical rotations of the isolated compounds were measured on a Jasco DIP-1000 digital polarimeter (Jasco Corporation, Tokyo, Japan). IR spectra were recorded on a Hitachi I-2001 infrared spectrophotometer (Hitachi High-Tech Corporation, Tokyo, Japan). The NMR spectra were recorded on a Bruker AVANCE-DPX 300 FT-NMR (Bruker, Bremen, Germany) at 300 MHz for ^1^H and 75 MHz for ^13^C in CDCl_3_, acetone-*d_6_*, CD_3_OD, DMSO-d_6_, or D_2_O (Sigma-Aldrich, St. Louis, USA) using signals of residual non-deuterated solvents as internal standards. Low-resolution mass spectral data were obtained by EI or FAB with a VG QUATTRO GC/MS spectrometer (VG Biotech, Altrincham, UK). HRMS spectral data were recorded by EI FT-MS on a BRUKER APEX II mass spectrometer. Silica gel 60 (230–400 mesh Merck, Darmstadt, Germany), Diaion^®^ HP-20P, MCI gel^®^ CHP20P (Mitsubishi Chemical Industries Ltd., Tokyo, Japan), Sephadex LH-20 (Pharmacia Fine Chemicals, Uppsala, Sweden); and precoated silica gel plates (Merck, Kieselgel 60 F254, 0.2 mm) were used for open CC and analytical TLC analysis, respectively. Isolation by NP-HPLC was performed by Shimadzu SPD-10A instrument (Shimadzu, Kyoto, Japan) equipped with an NP-column (Hibar Lichrosorb Si-60, 7 μm, 250 × 25 mm, Merck).

### 2.2. Animal Material

The Condy Anemone, *Condylactis* sp. (Weinland 1860), Actiniidae, was collected by hand via SCUBA were collected at 8~10 m depth from Tiao-Shi Reefs in Kenting at the southern tip of Taiwan, in April 2003, and stored in a freezer at −80 °C until extraction. A voucher sample (NHSC 2003-K5) was deposited at the Department of Marine Biotechnology and Resources, National Sun Yat-sen University.

### 2.3. Extraction and Separation

The sea anemone *Condylactis* sp. (1.0 kg, wet wt.) frozen bodies were sliced, homogenized in EtOH (6.0 L), and filtered off. The solvent-free crude extract was triturated in MeOH. The MeOH soluble portion (A1, 44.5 g) was fractionated on a column of Sephadex LH-20 eluted with MeOH (6 L) to yield three fractions A11 (orange semisolid residue, 19.0 g), A12 (reddish gummy residue, 24.5 g), and A13 (green solid mass, 0.9 g). Fraction A12, which exhibited an in vitro cytotoxicity, was chromatographed on a column of Si gel 60 using EtOAc–*n*-hexane (stepwise, 7.5–100% EtOAc) followed by MeOH–EtOAc (stepwise, 10–50% MeOH) to afford 24 subfractions A1201 to A1225. Fraction A1211 (139.5 mg) eluted with 100% EtOAc was purified by normal phase (NP)-HPLC using acetone—*n*-hexane (3:17) to yield **7** (6.5 mg), **8** (6.0 mg), **9** (8.3 mg), and **10** (2.1 mg), respectively. Fractions A1219 (139.5 mg) and A1220 eluted with 50% MeOH in EtOAc gave crude yellow solids which were recrystallized from acetone to afford the same compound **2** (113.5 and 36.7 mg yield, respectively). The acetone-soluble portion (20 mg) of fraction A1222 (175 mg), eluted with 50% MeOH in EtOAc, was isolated by NP-HPLC using acetone—CH_2_Cl_2_ (1:4) to give **3** (3 mg). Fraction A1224 (175 mg) was separated by NP-HPLC using EtOAc–MeOH–H_2_O (80:12:8) to give **5** (52.5 mg) and crude solid. The latter was further purified by NP-HPLC using EtOAc–MeOH–H_2_O (80:12:8) followed by an MCI gel^®^ CHP20P column using only H_2_O as an eluent to afford **1** (13.6 mg). Fraction A1225 (199 mg) was permeated through an MCI gel^®^ CHP20P column using H_2_O–MeOH (10:1 to 9:1, gradient) to produce **6** (9 mg). Fraction A11 was dissolved in 10% MeOH in distilled water, applied to the top of Diaion^®^ HP-20 column (1 L void volume), and eluted with a mixture of increasing percent of MeOH in distilled H_2_O to produce five fractions (A111–A115). A part (4.2 g) of A111 (13.3 g) eluted with 10% MeOH was purified by permeation through an MCI gel^®^ CHP20P column using distilled H_2_O followed by 5% MeOH in distilled H_2_O as an eluent to yield compound **4** (2.4 g).

#### 2.3.1. 3,5-Bis(3-pyridinyl)-1,2,4-thiadiazole (**1**)

Off-white powder; UV (MeOH) λ_max_ (log ε) 239 (6.84) and 291 (5.36) nm; IR (neat) ν_max_ 3057, 1589, 1560, 1478, 1402, 1339, 1296, 1128, 812, 698 cm^−1^; ^1^H and ^13^C NMR data in CDCl_3_, see Table 1; FABMS *m*/*z* 241 [100, (M + H)^+^], 105 [36.7, (C_6_H_4_N_2_ + H)^+^]; EIMS (70 eV) *m*/*z* 240 [19.8, (M)^+^], 162 [11.1, (M–C_5_H_4_N)^+^], 136 [100, (C_6_H_4_N_2_S)^+^], 122 [2.1, (C_6_H_4_NS)^+^], 104 [85.3, (C_6_H_4_N_2_)^+^], 78 [29.1, (C_5_H_4_N)^+^]; HREIMS *m*/*z* 240.0467 (calcd for C_12_H_8_N_4_S, 240.0469], 136.0089 (calcd for C_6_H_4_N_2_S, 136.0095], 104.0373 (calcd for C_6_H_4_N_2_, 104.0374].

#### 2.3.2. Thionicotinamide (**2**)

Pale yellow prisms (acetone); m.p. 188–189°; UV (MeOH) λ_max_ (log ε) 233 (19.08) and 296 (12.81) nm; IR (neat) ν_max_ 3229, 3026 (broad), 2844, 1678, 1570, 1452, 1402, 1311 cm^−1^; ^1^H and ^13^C NMR data in acetone-*d_6_* (see Table 1); FABMS *m*/*z* 139 [100, (M + H)^+^], 123 [7.9, (M–NH_2_ + H)^+^]; EIMS (30 eV) *m*/*z* 138 [100, (M)^+^], 122 [14.3, (M–NH_2_)^+^], 105 [80.9, (M–SH)^+^], 78 [70.9, (M–CSNH_2_)^+^]; HREIMS *m*/*z* 138.0243 (calcd for C_6_H_6_N_2_S, 138.0251], 122.0059 (calcd for C_6_H_4_NS, 122.0064], 105.0446 (calcd for C_6_H_5_N_2_, 105.0452]. The spectroscopic data were in agreement with those reported previously [26].

#### 2.3.3. Nicotinamide (**3**)

White powder; UV (MeOH) λ_max_ (log ε) 220 (9.83) and 261 (5.22) nm; IR (neat) ν_max_ 3414, 3214, 3004, 2924, 2824, 1682, 1622, 1387, 1262, 1098, 1030 cm^−1^; ^1^H and ^13^C NMR data acetone-*d_6_* (see Table 1); FABMS *m*/*z* 123 [100, (M + H)^+^], 107 [11.6, (M–NH_2_ + H)^+^]; EIMS (30 eV) *m*/*z* 122 [100, (M + H)^+^], 106 [70.9, (M–NH_2_)^+^], 78 [70.9, (M–CONH_2_)^+^], 51 (69.2). The NMR and MS spectroscopic data were in agreement with those reported previously [27].

#### 2.3.4. (−)-Betonicine (**4**)

Syrup; [α]^25^_D_ = −136.7 (c 0.3, H_2_O) (lit. −26.4, *c*.1.1, H_2_O [22]); UV (H_2_O) λ_max_ (log ε) 196 (43.00) and 329 (11.11) nm; IR (neat) ν_max_ 2000–3600 (broad), 1624 (broad), 1404 cm^−1^; ^1^H NMR (D_2_O, 300 MHz) δ_H_ 4.27 (1H, dd, *J* = 10.2, 7.5 Hz, H-2), 2.25 (1H, m, H-3α), 2.56 (1H, ddd, *J* = 17.4, 10.2, 7.5 Hz, H-3β), 4.60 (1H, m, H-4), 3.95 (1H, dd, *J* = 13.0, 6.3 Hz, H-5β), 3.42 (1H, dd, *J* = 13.0, 3.6 Hz, H-5α), 3.29 (3H, s, H_3_-6), 3.09 (3H, s, H_3_-7); ^13^C NMR (D_2_O, 75 MHz) δ_C_ 170.7 (C, C-7), 76.3 (CH, C-2), 74.1 (CH_2_, C-5), 66.5 (CH, C-4), 54.5 (CH_3_, C-6), 48.7 (CH_3_, C-7), 36.1 (CH_2_, C-3); EIMS (70 eV) *m*/*z* 160 [0.7, (M)^+^], 100 [48.3, (M–CO_2_–CH_4_)^+^], 82 [43.4, (M–CO_2_–H_2_O–CH_4_)^+^], 67 [5.7, (M–CO_2_–H_2_O–CH_3_–CH_4_)^+^].

#### 2.3.5. Uridine (**5**)

White powder; mp 163-164; UV (MeOH) λ_max_ (log ε) 217 (2.62) and 253 (3.20) nm; IR (KBr) ν_max_ 3396 (br s), 2986, 2926, 1682, 1469, 1397, 1269, 1209, 1098, 1053 cm^−1^; ^1^H NMR (CD_3_OD, 300 MHz) δ 08.00 (1H, d, *J* = 8.1 Hz, H-6), 5.89 (1H, d, J = 4.4 Hz, H-1′), 5.69 (1H, d, *J* = 8.1 Hz, H-5), 4.17 (1H, dd, *J* = 4.4, 5.0 Hz, H-2′), 4.14 (1H, dd, *J* = 6.0, 5.0 Hz, H-3′), 4.00 (1H, dd, *J* = 6.0, 3.3 Hz, H-4′), 3.83 (1H, dd, *J* = 12.0, 3.3 Hz, H-5′a), 3.70 (1H, dd, *J* = 12.0, 3.3 Hz, H-5′b); ^13^C NMR: δ 166.2 (C, C-4), 152.5 (C, C-2), 142.7 (CH, C-6), 102.7 (CH, C-5), 90.7 (CH, C-1′), 86.4 (CH, C-4′), 75.7 (CH, C-2′), 71.3 (CH, C-3′), 62.3 (CH_2_, C-5′); EIMS (30 eV) *m*/*z* 244 [0.4, (M)^+^], 226 [5.6, (M–H_2_O)^+^], 133 [42.1, (C_5_H_9_O_4_)^+^], 113 (100). The NMR and MS spectroscopic data were in agreement with those reported previously [28].

#### 2.3.6. Hypoxanthine (**6**)

White powder; ^1^H NMR (DMSO-*d_6_*, 300 MHz) δ 7.95 (1H, s), 8.08 (1H, s); ^13^C NMR (DMSO-*d_6_*, 75 MHz) δ 155.7 (C), 153.6 (C), 144.7 (CH), 140.6 (CH), 119.5 (C); FABMS *m*/*z* 137 [11.7, (M + H)^+^], EIMS (70 eV) *m*/*z* 136 [33, (M)^+^], 100 (53.4), 82 (29.2), 66 (21.7). Data is in agreement with those in SDBS [26].

#### 2.3.7. 5α,8α-Epidioxy-24S-methylcholest-6-en-3β-ol (**7**)

White amorphous solid. MS, ^1^H, and ^13^C NMR data in CDCl_3_ were found to be in full agreement with those reported previously [29].

#### 2.3.8. 5α,8α-Epidioxy-24R-methylcholesta-6,22E-dien-3β-ol (**8**)

White amorphous solid. MS, ^1^H, and ^13^C NMR data in CDCl_3_ were found to be in full agreement with those reported previously [30,31,32].

#### 2.3.9. 5α,8α-Epidioxy-24-methylenecholesta-6-en-3β-ol (**9**)

White amorphous solid. MS, ^1^H, and ^13^C NMR data in CDCl_3_ were found to be in full agreement with those reported previously [32].

#### 2.3.10. (22R,23R,24R)-5α,8α-Epidioxy-22,23-methylene-24-methylcholesta-6-en-3β-ol (**10**)

White amorphous solid. MS, ^1^H, and ^13^C NMR data in CDCl_3_ were found to be in full agreement with those reported previously [33].

### 2.4. Cytotoxicity Testing

The cancer cell lines: NCI-H661 (human lung carcinoma), KB (human oral epidermoid carcinoma), Hepa59T/VGH (human liver carcinoma), HeLa cells (human cervical cancer), and Med (human medulloblastoma) were purchased from the American Type Culture Collection (ATCC). Cytotoxicity assays of the substances were performed using MTT colorimetric method [34]. The compound is considered inactive when IC_50_ > 20 μg/mL. The positive control used is mitomycin.

## 3. Results and Discussion

**Figure 1:**
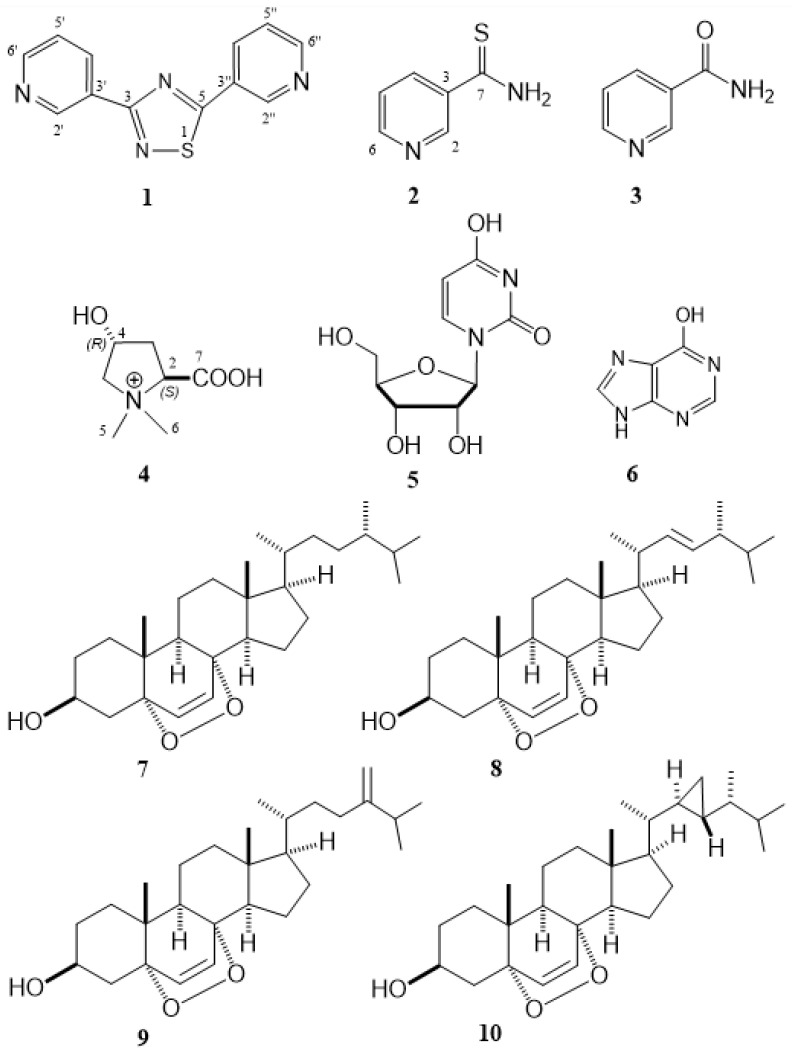
Metabolites isolated from the anemone *Condylactis* sp.

The frozen bodies of *Condylactis* sp. were sliced and extracted with ethanol (EtOH). The solvent-free crude extract was triturated in methanol (MeOH). The MeOH-soluble portion was then fractionated on Sephadex LH-20 to yield three fractions. A fraction that exhibited an in vitro cytotoxicity against Hepa59T/VGH and HeLa cells (IC_50_ 18.9 and 18.3 μg/mL, respectively), was subjected to chromatographic isolation using a combination of a normal phase (NP)-HPLC and MCI gel^®^ CHP20P columns to yields compounds **1**–**3**, **5**–**10**. Moreover, columns of Diaion^®^ HP-20P and MCI gel^®^ CHP20P were also utilized to obtain compound **4** from a major non-cytotoxic fraction MeOH-soluble fraction. The molecular structures of **1**–**10** (Figure 1) were established mainly by MS and NMR spectroscopic analyses (Table 1 and Supplementary Figures S1–S27), and by comparison of their NMR data with those in the literature and databases. The four epidioxysteroids, among other metabolites, showed significant cytotoxic activities against five cancer cell lines (Table 2).

**Figure 2:**
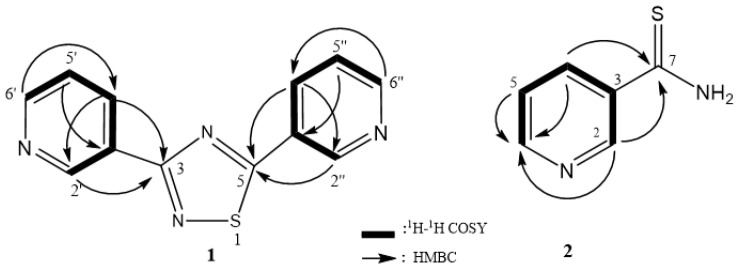
^1^H-^1^H COSY and HMBC correlations of **1** and **2**.

**Figure 3:**
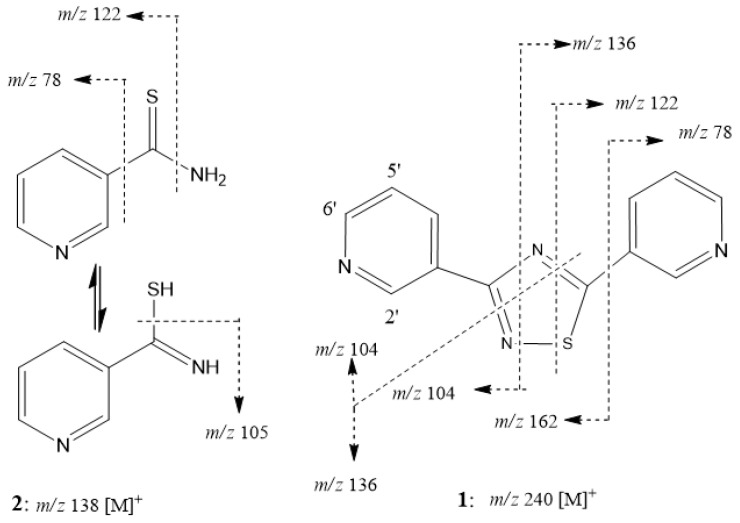
EI-MS fragmentation pattern of **1** and **2**.

**Scheme 1:**
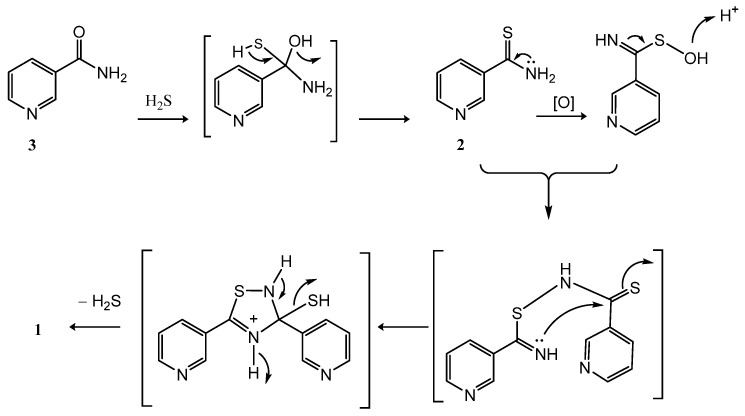
Proposed biosynthetic pathway of **1**.

Compound **1** was obtained as an off-white powder. Its molecular ion peak at *m*/*z* 240.0467 in the high-resolution electron impact mass spectrometry (HREIMS) indicated a molecular formula of C_12_H_8_N_4_S and eleven degrees of unsaturation. The ^13^C NMR data revealed the presence of two pyridinyl groups, equivalent to eight unsaturation degrees, from the two sets of five carbon signals (C-2′ to C-6′ and C-2″ to C-6″) appearing from δ_C_ 153.0 to 123.9 as those of the co-isolated compound **2** (Table 1). This was further supported by the ion peaks that appeared at *m*/*z* 162 in EIMS after the elimination of a pyridinyl (C_5_H_4_N)^+^ group. Therefore, the remaining two sp^2^ quaternary carbon signals at δ_C_ 171.4 and 185.8 should be incorporated in a diunsaturated heterocyclic ring having one sulfur and two nitrogen atoms to form a thiadiazole core. The appearance of twelve ^13^C signals in the molecule of **1** instead of six omits the possibility of the presence of the symmetrical 1,2,5- or 1,3,4-thiadiazol as a linking unit for **1**. The analysis of the COSY) and HMBC spectra (Figure 2) established the structure of compound **1** as a 1,2,4-thiadiazole-based alk1aloid. The *^3^J_CH_* correlations of the two sets of pyridinyl protons (H-2′/H-4′ and H-2″/H-4″) to carbons C-3 (δ_C_ 171.4, C) and C-5 (δ_C_ 185.8, C) designated linking the position of pyridinyl groups to C-3 and C-5 of thiadiazole ring, respectively. Moreover, a fission of the molecule into [C_6_H_4_N_2_]^+^ at *m*/*z* 104 and [C_6_H_4_N_2_S]^+^ at *m*/*z* 136 was observed in the EIMS of **1** (Figure 3). The latter fragment ion further yielded [C_6_H_4_NS]^+^ at *m*/*z* 122 after the loss of one nitrogen atom. This fragmentation pattern and others could further support the identity of metabolite **1** as 3,5-bis(3-pyridinyl)-1,2,4-thiadiazole, but not 3,5-bis(3-pyridinyl)-1,2,3-thiadiazole. This 1,2,4-thiadiazole-based alkaloidal structure of compound **1** could be formed via oxidative dimerization of thioniacinamide (**2**). This dimerization process could be initialized in nature by the formation of thioamide *S*-oxide [35] after acidic oxidation of **2** or in the presence of an electrophilic environment [36]. Consequently, a plausible biosynthetic pathway of **1** from **2** was suggested as shown in Scheme 1 and further established the formation of the 1,2,4-thiadiazole moiety, but neither 1,2,3-, 1,3,4-, nor 1,2,5-thiadiazole, as a linking unit in the molecule of **2**. Finally, a comparison of the NMR data of compound **2** with those of a synthetic product prepared by copper(II)-mediated homocoupling of thioamides revealed the same chemical identity [37]. However, the full assignment of ^1^H and ^13^C NMR signals was reported herein by the analysis of 2D NMR correlations for the first time. Despite the fact that the non-pyridinyl substituted-1,2,4-thiadiazoles are very rare, being only reported in nature from a fungus [38], a plant [39], and an ascidian [40], this is the first report to isolate a 3,5-bispyridinyl-substituted 1,2,4-thiadiazole (**1**) as a new type of alkaloid from nature.

Compound **2** was obtained as pale yellow prisms and possessed a molecular formula of C_6_H_6_N_2_S from its HREIMS (*m*/*z* 138.0243). Its IR absorption bands at ν_max_ 3229 and 3026 (broad) cm^−1^ along with characteristic absorptions at 1678, 1452, and 1311 cm^−1^, suggesting the presence of aromatic or heteroaromatic thioamides [41,42]. The ion peaks displayed in the EIMS at *m*/*z* 122 [M–NH_2_]^+^ and 78 [C_5_H_4_N]^+^ indicated that the remaining C and S atoms should be consistent with a thiocarbonyl functionality to fulfill the fifth unsaturation degree in the molecule. The four downfield shifted protons at δ_H_ 7.42–9.11 together with the four sp^2^ methine (δ_C_ 123.7–152.5, 4C) and the two sp^2^ quaternary (δ_C_ 136.3 and 200.4, 2C) carbons (Table 1) in the ^13^C NMR spectrum and the analysis of 2D NMR spectral correlations (Figure 2) were thus in a good agreement for a 3-pyridine-carbothioamide or thionicotinamide (**2**). To the best of our knowledge, compound **2** is a natural product isolated for the first time from a living organism. This compound might be derived from thionation of the co-existing nicotinamide (**3**) [43] through a reaction with H_2_S from the conversion of a sulfur source of the ocean by sulfate-reducing bacteria [44] or similar symbiotic bacteria associated with sea anemones [45,46] (Scheme 1).

The present study also led to the isolation of nicotinamide (**3**), which showed similar ^1^H and ^13^C signals to those of **2**, except for those of carbonyl (δ_C_ 167.6, C) and thiocarbonyl (δ_C_ 200.4, C) carbons, respectively. This is further confirmed by direct mass and NMR data comparison with those previously reported [27].

**Figure 4:**
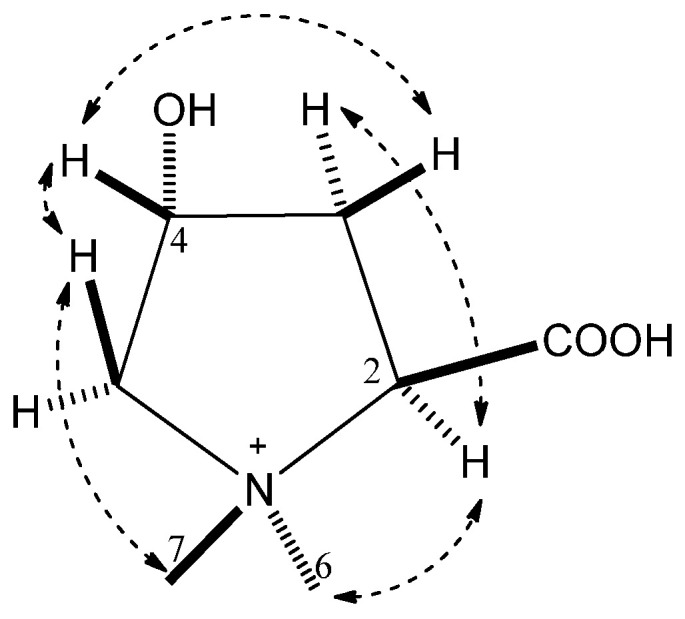
NOE correlation of compound **4**.

The water-soluble compound (−)-betonicine (**4**) was established to have a molecular formula of C_7_H_14_NO_3_ from its molecular ion peak at *m*/*z* 160 [M]^+^ in the EIMS and NMR data (see experimental section). Moreover, the ion peaks appearing at *m*/*z* 100 [M–CO_2_–CH_4_]^+^, 82 [M–CO_2_–H_2_O–CH_4_]^+^, 67 [M–CO_2_–H_2_O–CH_3_–CH_4_]^+^ in the EIMS pointed out the presence of one carboxylic, one hydroxymethine group, and two *N*-methyl groups in the molecule of **4**. This is further substantiated by the NMR signals measured in D_2_O at δ_C_/δ_H_ 170.7 (C), 66.5 (CH)/4.60 (1H, m), 48.7 (CH_3_)/3.09 (3H, s), and 54.5(CH_3_)/3.29 (3H, s), respectively. The 2D NMR spectroscopic analysis established **4** as *N*,*N*′-dimethyl-4-hydroxyproline whereas the analysis of NOE correlations (Figure 4) proved the *2S* and *4R* configuration in the structure of **4** which was found to be the same as (−)-betonicine [47] that has been found in higher plants, e.g., *Melaleuca* spp. [47]. However, the isolation of (−)-betonicine, a pyrrolidine alkaloid, from a marine invertebrate is reported herein for the first time (based on the sign of optical activity; [α]^25^_D_ = −136.7, *c* 0.3, H_2_O) although the enantiomeric (+)-betonicine ([α]^25^_D_ = +15.5, *c* 0.3, H_2_O) has been isolated from a marine red algae *Ahnfeltiopsis flabelliformis* [48]. The full assignment of NMR data of **4** is presented herein for the first time at neutral pH (c.f. previous NMR data measured at different acidic pH [47]).

In addition to the isolated compounds **1**–**4**, other known metabolites (**5**–**10**) were also obtained from the cytotoxic MeOH-soluble fraction of the anemone ethanolic extract (Figure 1). On the basis of the spectroscopic analyses and by comparison of physical and spectroscopic (IR, MS, and NMR) data with those reported, compounds **5**–**10** were identified as uridine (**5**) [28], hypoxanthine (**6**) [26], 5α,8α-epidioxy-24*S*-methylcholest-6-en-3β-ol (**7**) [29], 5α,8α-epidioxy-24*R*-methylcholesta-6,22*E*-dien-3β-ol (**8**) [30,31], 5α,8α-epidioxy-24-methylenecholesta-6-en-3β-ol (**9**) [32], and (22*R*,23*R*,24*R*)-5α,8α-epidioxy-22,23-methylene-24-methylcholesta-6-en-3β-ol (**10**) [33].

The heterocyclic compounds **1**–**5** were shown to be non-cytotoxic (IC_50_ > 20 μg/mL) against the growth of cancer cell lines such as NCI-H661 (human lung carcinoma), KB (human oral epidermoid carcinoma), Hepa59T/VGH (human liver carcinoma), HeLa (human cervical cancer), and Med (human medulloblastoma) cells. However, it has been reported that compound **1** has much stronger inhibitory activity against aromatase than resveratrol and thus could be served as an effective non-steroidal chemopreventive candidate in the treatment of estrogen-sensitive breast cancer [49]. Thionicotinamide (**2**) is considered an inhibitor for nicotinamide adenine dinucleotide kinase (NADK) [50], metabolic targets controlling redox co-enzymes. Therefore, although compound **2** displayed no cytotoxicity against the tested cancel lines in this study, a mixture of thionicotinamide (**2**) with NADK inhibitors might represent an efficacious antitumor combination as it reduces nicotinamide adenine dinucleotide phosphate (NADPH) pools and accelerates the degradation of dihydrofolate reductase (DHFR), synergistically inhibiting the proliferation of cancer cells [51,52]. In addition, a combination treatment of thionicotinamide adenine dinucleotide phosphate (NADPS) with methotrexate (MTX) demonstrated significant synergy in a metastatic colon cancer cell line and was effective in an MTX-resistant leukemic cells [52]. Although the thionicotinamide therapy in tuberculosis is less-effective relative to rifampicin or isoniazid, the use of thioamides such as **2** can overcome multidrug-resistant tuberculosis [53,54]. A pyridine-4-thiocarboxymide analog, ethionamide, a second-line anti-tubercular drug, can be activated by enzymatic oxidation to exert the pharmaceutical effect [55]. A mimicked redox process involving electron transfer reaction of hexacyanoferrate (III) and thionicotinamide (**2**) also showed the potential for the design of new thioamide-containing medicines with the aim of fighting the drug-resistant strains of *Mycobacterium tuberculosis* [53].

The pyrrolidine alkaloid (**4**) has been shown an unprecedented stimulatory effect similar to *N*-octanoyl-DL-homoserine lactone (OHL) on bacterial quorum sensing (QS), a clue that could be useful for the development of potential QS inhibitors which are chemically unrelated to acylated homoserine lactones (AHLs) [48]. Therefore, the anemone under investigation is regarded as a rich source for this medicinally important natural product (**4**), particularly when it could be cultivated.

The acetylated product of **5**, uridine triacetate, has been shown to be an effective antidote against life-threatening toxicity and mortality resulting from overdoses or exaggerated susceptibility to the widely used anticancer drugs 5-fluorouracil (5-FU) and capecitabine [56]. It has been approved by FDA since 2018.

The isolated 5α,8α-epidioxy sterols (**7**–**10**) were also evaluated for their cytotoxic potential and were found to possess strong to moderate activity (IC_50_ 3.5–9.0 μg/mL) against Hepa59T/VGH, NCI-H661, KB, HeLa, and Med cancer cells (Table 2).

Terpenoid and steroid peroxides isolated from terrestrial and marine organisms represent an interesting bioactive group of natural products [57,58,59,60]. These natural peroxides have been reported to exhibit a variety of biological activities, which may be linked to their ability to release oxygen, such as antiprotozoal (e.g., antimalarial) [61,62], anthelmintic, antiviral [63,64], and cytotoxic [33,58,65,66] activities. Moreover, the 5α,8α-epidioxy sterol **8**, which was also isolated from edible mushrooms, has also been reported to possess anti-tumor activity against breast cancer (MCF-7) [65,66] and carcinosarcoma (Walker 256) cells [65] and to act as an inhibitor against 12-*O*-tetradecanoylphorbol-13-acetate (TPA)-induced tumor promotion in mouse skin [67], immunosuppressant [68], and anti-inflammatory [69]. Compounds **7** and **9** extended their cytotoxic property to be also against MCF-7 cancer cells [66]. Furthermore, the cyclopropane possessing 5α,8α-epidioxy sterol (**10**) has been shown to exert potent cytotoxicity against other cancer cell lines such as MCF-7, HEp 2 (laryngeal adenocarcinoma), and DLD-1 (human colorectal adenocarcinoma) cancer cells [70]. Furthermore, **10** also showed the anti-inflammatory effect as it effectively downregulated the in vitro LPS-induced iNOS expression [70]. However, it seems that the 5α,8α-epidioxy sterols have certain selective inhibitory activity against a variety of cancer cells, as compound **8** or its 24-epimer, which did not exhibit cytotoxicity against certain cancer cells such as human erythroleukemia (K-562), lymphoid T carcinoma (MOLT-4), and (DLD-1) [60]. Although the thio-metabolites (**1** and **2** did not show cytotoxic activity, future biological screening and evaluation are still required.

## 4. Conclusions

The organic soluble extract of the invasive sea anemone *Condylactis* sp. was chemically investigated for the first time, isolating several S-, N-, and O-heterocyclic metabolites. The 3,5-bis(3-pyridinyl)-1,2,4-thiadiazole alkaloid and its biosynthetic precursor thionicotinamide were recognized herein as natural products discovered for the first time from nature. Moreover, four 5,8-epidioxysteroid metabolites were isolated and significantly inhibited the growth of a limited panel of cancer cell lines and were considered responsible for the cytotoxicity of their organic soluble fraction. (−)-Betonicine was also separated from marine organisms as a major polar N-heterocyclic metabolite for the first time. Aside from the proteinaceous venom of anemones of the genus *Condylactis*, other organic soluble metabolites are worthy of being studied with the aim of discovering molecules of chemical and pharmaceutical interest. A massive harvest of this *Condylactis* anemone, invasive to coral reefs, is not only beneficial to the marine reef ecosystem but also makes this environmentally harmful organism a valuable resource of biologically active compounds.

## Figures and Tables

**Table 1:** ^1^H and ^13^C NMR data of compounds **1**–**3** in CDCl_3_.

	1			2		3	
Atom	δ_C_	δ_H_	Atom	δ_C_	δ_H_	δ_C_	δ_H_
3		171.4 (C)					
5		185.8 (C)	7		200.4 (C)		167.6 (C)
2′	9.63 1H, br d (1.8) *^a^*	149.2 (CH) *^b^*	2	9.11 1H, d (1.6)	148.5 (CH)	9.08 1H, s	149.8 (CH)
3′		128.8 (C)	3		136.3 (C)		130.8 (C)
4′	8.70 1H, ddd (7.9, 1.8, 1.8)	136.2 (CH)	4	8.27 1H, br d (7.8)	135.5 (CH)	8.22 1H, d (7.2)	135.9 (CH)
5′	7.52 1H, ddd (7.3, 5.0, 1.8)	123.9 (CH)	5	7.42 1H, dd (7.8, 5.0)	123.7 (CH)	7.45 1H, dd (7.2, 5.0)	124.2 (CH)
6′	8.75 1H, dd (5.0, 1.8)	150.6 (CH)	6	8.66 1H, d (5.0)	152.5 (CH)	8.68 1H, d (5.0)	153.0 (CH)
2″	9.28 1H, br d (1.8)	148.6 (CH)					
3″		126.7 (C)					
4″	8.37 1H, ddd (7.9, 1.8, 1.8)	134.6 (CH)					
5″	7.52 1H, ddd (7.3, 5.0, 1.8)	124.2 (CH)					
6″	8.81 1H, dd (4.9, 1.8)	153.0 (CH)					

Spectra recorded at 300 MHz for ^1^H and 75 MHz for ^13^C at 25 °C. *^a^* The *J* values are in Hz in parentheses. *^b^* Attached protons were determined by DEPT experiments. The δ values are in ppm downfield from TMS.

**Table 2:** Cytotoxicity (IC_50_ μg/mL) of fraction A12 and metabolites **1**–**10** of *Condylactis* sp.

Compound	Hepa 59T/VGH	NCI-H661	KB	HeLa	Med
Fraction A12	18.9	− *^a^*	>20	18.3	>20
1–5	>20	>20	>20	>20	>20
6	− *^a^*	− *^a^*	− *^a^*	− *^a^*	− *^a^*
7	7.4	6.0	7.6	4.7	7.5
8	8.8	7.1	9.0	8.4	7.7
9	7.0	5.8	3.5	5.7	5.7
10	6.5	6.6	7.9	6.1	4.2
Mitomycin	0.8	0.6	0.7	0.8	0.8

Fraction/compound is considered inactive when IC_50_ > 20 μg/mL. *^a^* Not tested.

## Data Availability

Data is contained within the article or Supplementary Materials.

## Supplementary Materials

The following supporting information can be downloaded at: https://www.mdpi.com/article/10.3390/metabo13030392/s1, MS; ^1^H and ^13^C NMR of compounds **1**–**10**; and 2D NMR spectra of compound **1**. Figure S1: HRESIMS spectrum of **1** (M^+^ and major ion peaks), Figure S2: EIMS spectrum of **1**, Figure S3: ^1^H NMR spectrum of **1** in CDCl_3_ at 300 MHz, Figure S4: ^13^C NMR spectrum of **1** in CDCl_3_ at 75 MHz, Figure S5: ^1^H-^1^H COSY spectrum of **1** in CDCl_3_, Figure S6: HSC spectrum of **1** in CDCl_3_, Figure S7: HMBC spectrum of **1** in CDCl_3_, Figure S8: HREIMS spectrum of **2** (M^+^ and major ion peaks), Figure S9: EIMS spectrum of **2**, Figure S10: ^1^H NMR spectrum of **2** in acetone-*d_6_* at 300 MHz, Figure S11: ^13^C NMR spectrum of **2** in acetone-*d_6_* at 75 MHz, Figure S12: EIMS spectrum of **3**, Figure S13: ^1^H NMR spectrum of **3** in acetone-*d_6_* at 300 MHz, Figure S14: ^13^C NMR spectrum of **3** in acetone-*d_6_* at 75 MHz, Figure S15: EIMS spectrum of **4**, Figure S16: ^1^H NMR spectrum of **4** in D_6_O at 300 MHz, Figure S17: ^13^C NMR spectrum of **4** in D_2_O at 75 MHz, Figure S18: EIMS spectrum of **5**, Figure S19: ^1^H NMR spectrum of **5** in CD_3_OD at 300 MHz, Figure S20: ^13^C NMR spectrum of **5** in CD_3_OD at 75 MHz, Figure S21: FABMS spectrum of **6**_,_ Figure S22: ^1^H NMR spectrum of **6** in DMSO-*d_6_* at 300 MHz, Figure S23: ^13^C NMR spectrum of **6** in DMSO-*d_6_* at 75 MHz, Figure S24: ^1^H NMR spectrum of **7** in CDCl_3_ at 300 MHz, Figure S25: ^1^H NMR spectrum of **8** in CDCl_3_ at 300 MHz, Figure S26: ^1^H NMR spectrum of **9** in CDCl_3_ at 300 MHz, Figure S27: ^1^H NMR spectrum of **10** in CDCl_3_ at 300 MHz.
