# Supplementary material for: The Invasive Anemone Condylactis sp. of the Coral Reef as a Source of Sulfur- and Nitrogen-Containing Metabolites and Cytotoxic 5,8-Epidioxy Steroids
Source: Metabolites. 2023 Mar 7;13(3):392. doi: 10.3390/metabo13030392 (PMC10056678; doi:10.3390/metabo13030392)
Supplement: Supplementary file 1 [file metabolites-13-00392-s001.zip › metabolites-2248589 - Supplementary Material.pdf]

Supplementary Materials:

The Invasive Anemone Condylactis sp. of the Coral Reef as a Source of Sulfur- and Nitrogen-containing Metabolites and Cytotoxic 5,8-Epidioxy Steroids.

Atallah F. Ahmed[1,2,3], Chang-Feng Dai[4], Yao-Haur Kuo[5], Jyh-Horng Sheu[1,6,7,8,9,*]

[1] Department of Marine Biotechnology and Resources, National Sun Yat-Sen University, Kaohsiung 804, Taiwan, Republic of China
[2] Department of Pharmacognosy, College of Pharmacy, King Saud University, Riyadh 11451, Saudi Arabia
[3] Department of Pharmacognosy, Faculty of Pharmacy, Mansoura University, Mansoura 35516, Egypt
[4] Institute of Oceanography, National Taiwan University, Taipei 106, Taiwan
[5] Division of Herbal Drugs and Natural Products, National Research Institute of Chines Medicine, Taipei 112, Taiwan
[6] Institute of Natural Products, Kaohsiung Medical University, Kaohsiung 807, Taiwan
[7] Department of Medical Research, China Medical University Hospital, China Medical University, Taichung 404, Taiwan
[8] Frontier Center for Ocean Science and Technology, National Sun Yat-sen University, Kaohsiung 804, Taiwan
[9] Doctoral Degree Program in Marine Biotechnology, National Sun Yat-sen University and Academia Sinica, Kaohsiung 804, Taiwan

List of Supplementary material

No.	Content	Page
**Figure S1**	HREIMS spectrum of **1** (M^+ and major ion peaks)	S3
**Figure S2**	EIMS spectrum of **1**	S4
**Figure S3**	^{1}H NMR spectrum of **1** in $CDCl_3$ at 300 MHz	S4
**Figure S4**	^{13}C NMR spectrum of **1** in $CDCl_3$ at 75 MHz	S5
**Figure S5**	^{1}H-^{1}H COSY spectrum of **1** in $CDCl_3$	S5
**Figure S6**	HSQC spectrum of **1** in $CDCl_3$	S6
**Figure S7**	HMBC spectrum of **1** in $CDCl_3$	S6
**Figure S8**	HREIMS spectrum of **2** (M^+ and major ion peaks)	S7
**Figure S9**	EIMS spectrum of **2**	S8
**Figure S10**	^{1}H NMR spectrum of **2** in acetone-d_6 at 300 MHz	S8
**Figure S11**	^{13}C NMR spectrum of **2** in acetone-d_6 at 75 MHz	S9
**Figure S12**	EIMS spectrum of **3**	S9
**Figure S13**	^{1}H NMR spectrum of **3** in acetone-d_6 at 300 MHz	S10
**Figure S14**	^{13}C NMR spectrum of **3** in acetone-d_6 at 75 MHz	S10
**Figure S15**	EIMS spectrum of **4**	S11
**Figure S16**	^{1}H NMR spectrum of **4** in D_2O at 300 MHz	S11
**Figure S17**	^{13}C NMR spectrum of **4** in D_2O at 75 MHz	S12
**Figure S18**	EIMS spectrum of **5**	S12
**Figure S19**	^{1}H NMR spectrum of **5** in CD_3OD at 300 MHz	S13
**Figure S20**	^{13}C NMR spectrum of **5** in CD_3OD at 75 MHz	S13
**Figure S21**	FABMS spectrum of **6**	S14
**Figure S22**	^{1}H NMR spectrum of **6** in DMSO-d_6 at 300 MHz	S14
**Figure S23**	^{13}C NMR spectrum of **6** in DMSO-d_6 at 75 MHz	S14
**Figure S24**	^{1}H NMR spectrum of **7** in $CDCl_3$ at 300 MHz	S15
**Figure S25**	^{1}H NMR spectrum of **8** in $CDCl_3$ at 300 MHz	S15
**Figure S26**	^{1}H NMR spectrum of **9** in $CDCl_3$ at 300 MHz	S16
**Figure S27**	^{1}H NMR spectrum of **10** in $CDCl_3$ at 300 MHz	S16

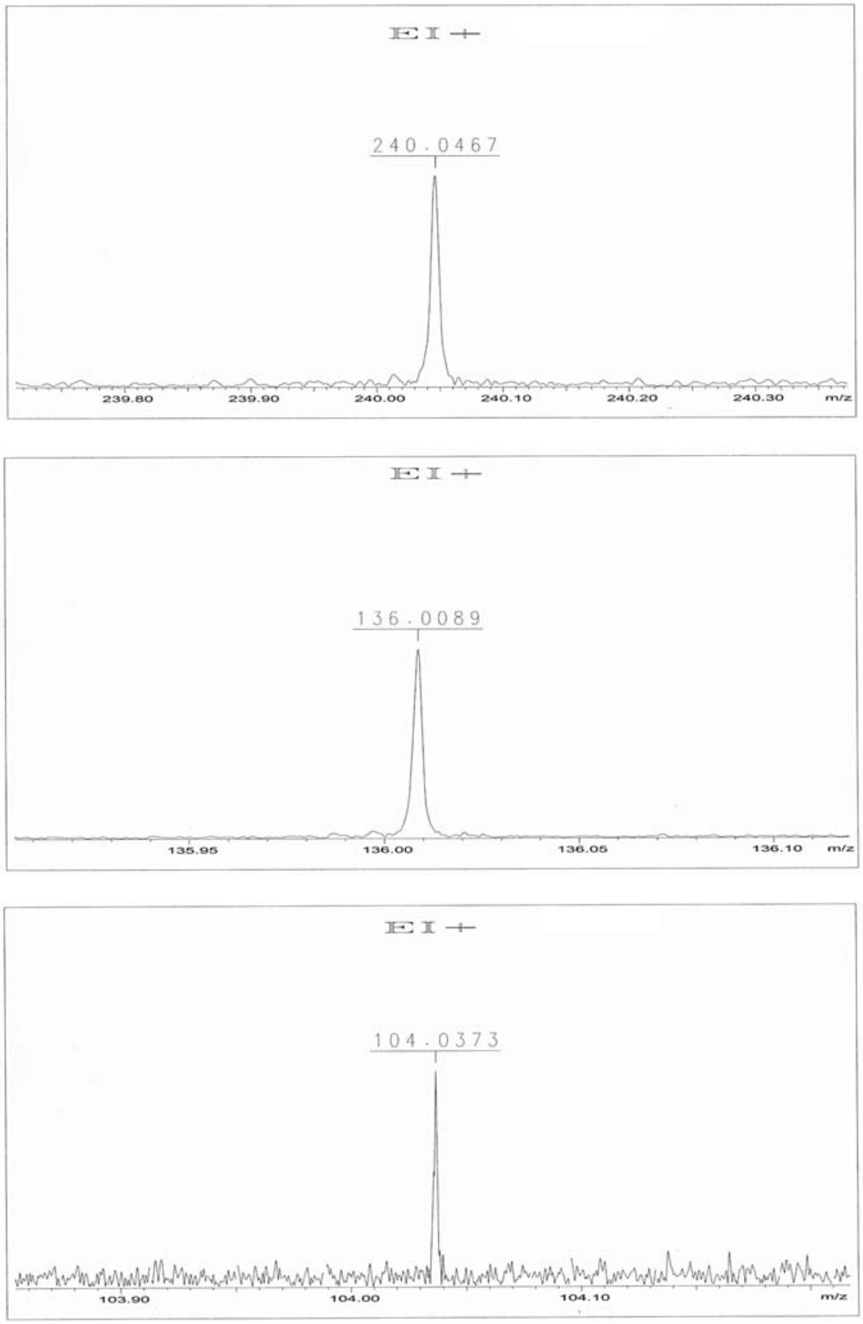

Figure S1. HREIMS spectrum of 1 (M^+ and major ion peaks)

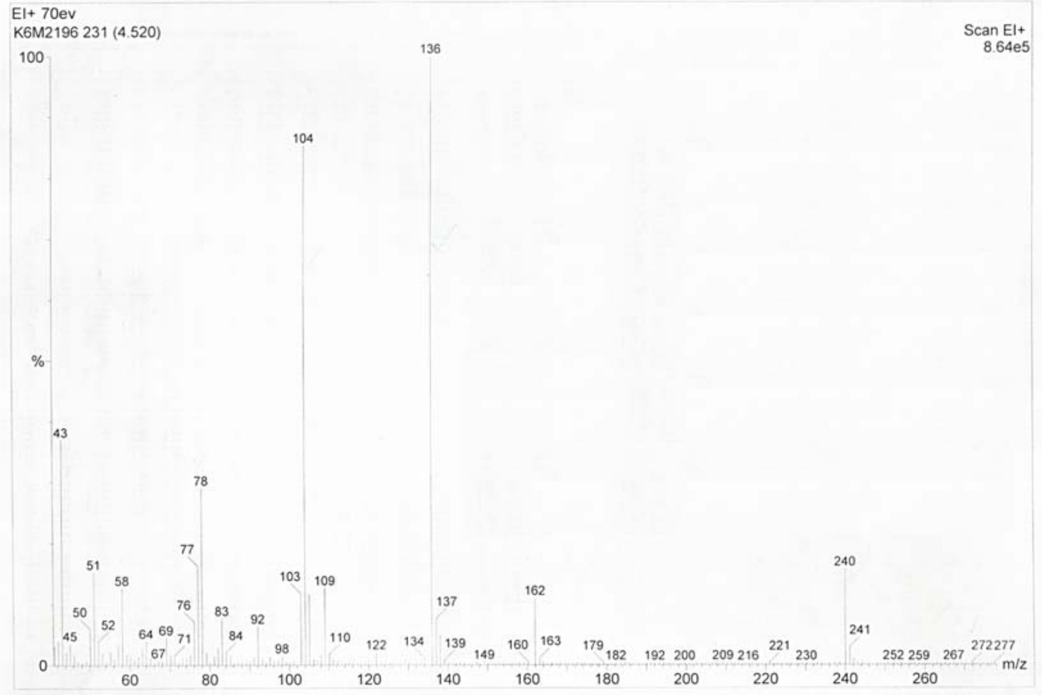

Figure S2. EIMS spectrum of 1

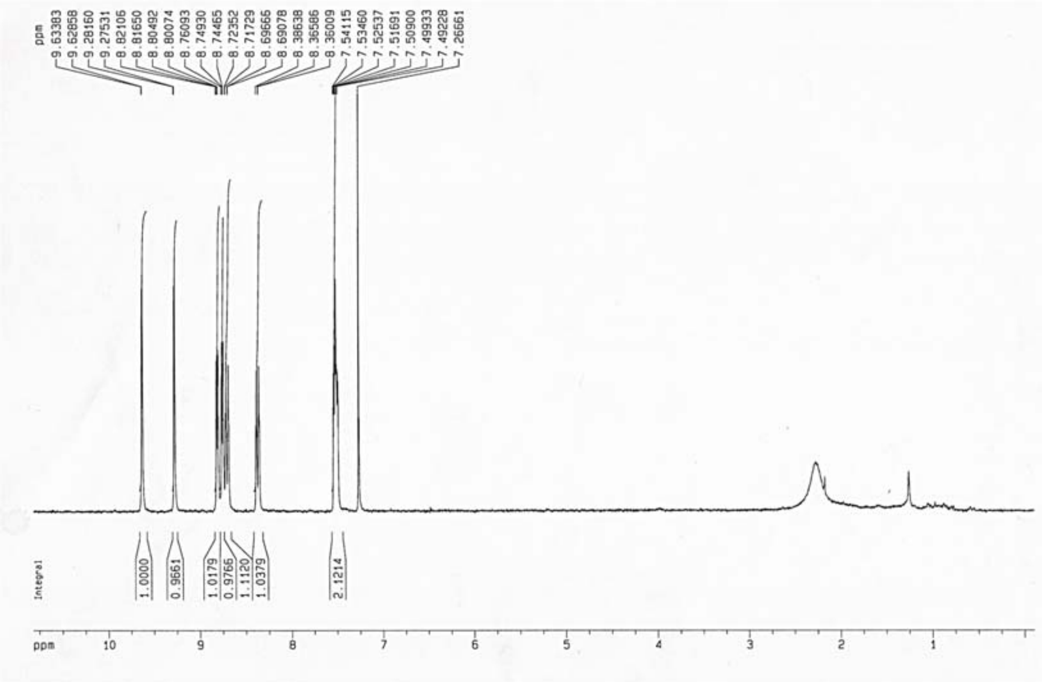

Figure S3. ^{1}H NMR spectrum of **1** in $CDCl_3$ at 300 MHz

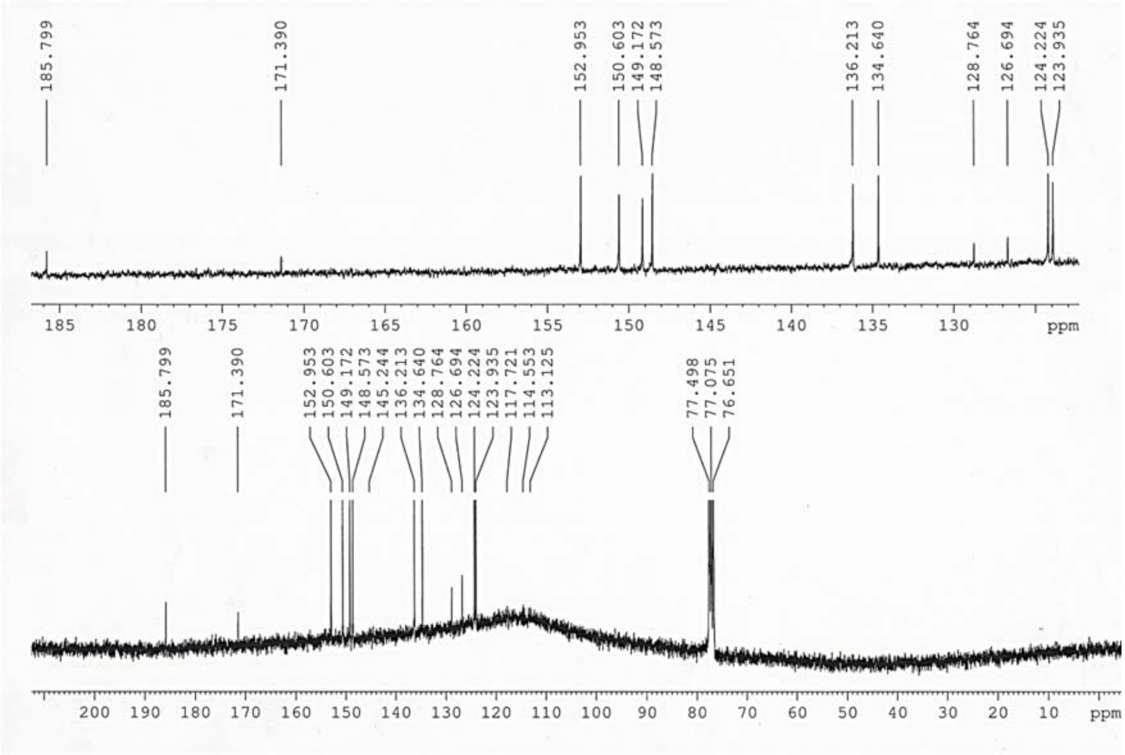

Figure S4. ^{13}C NMR spectrum of **1** in $CDCl_3$ at 75 MHz

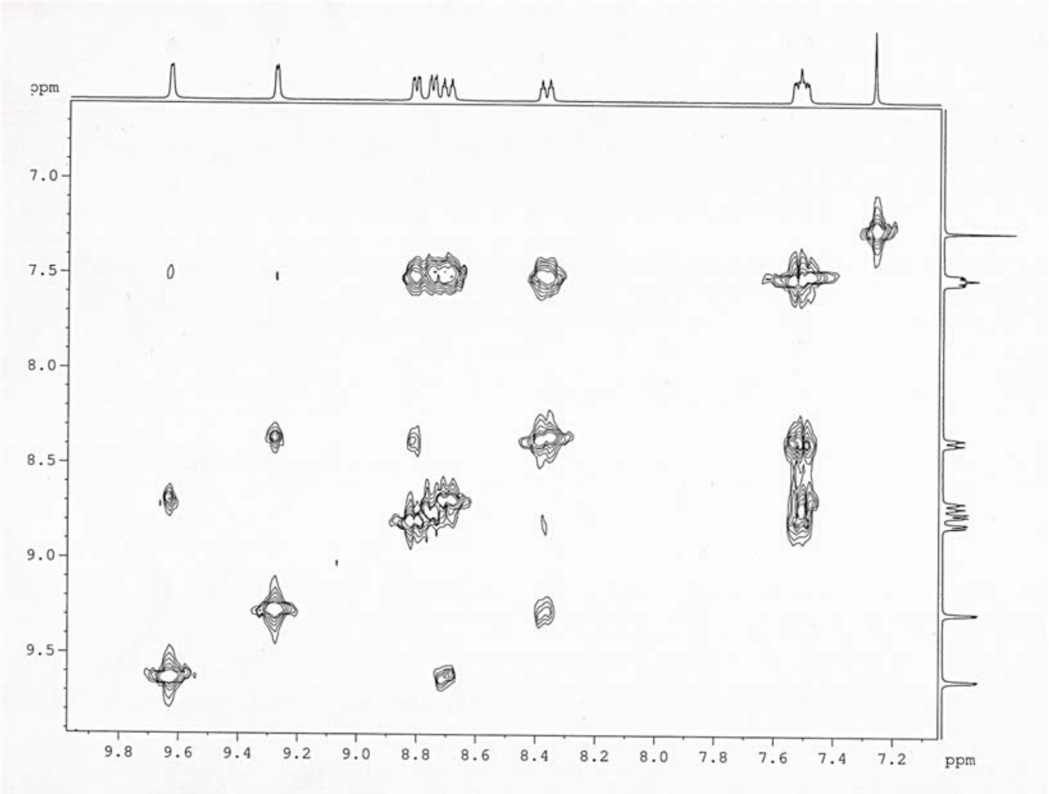

Figure S5. ^{1}H-^{1}H COSY spectrum of **1** in $CDCl_3$

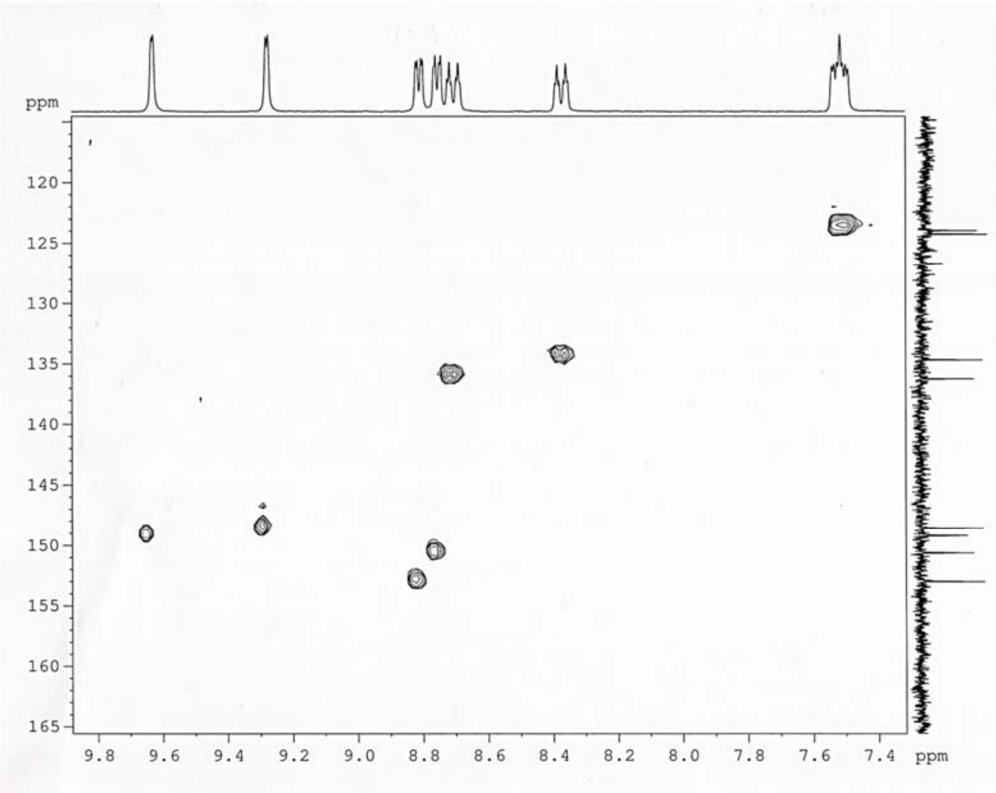

Figure S6. HSQC spectrum of **1** in $CDCl_3$

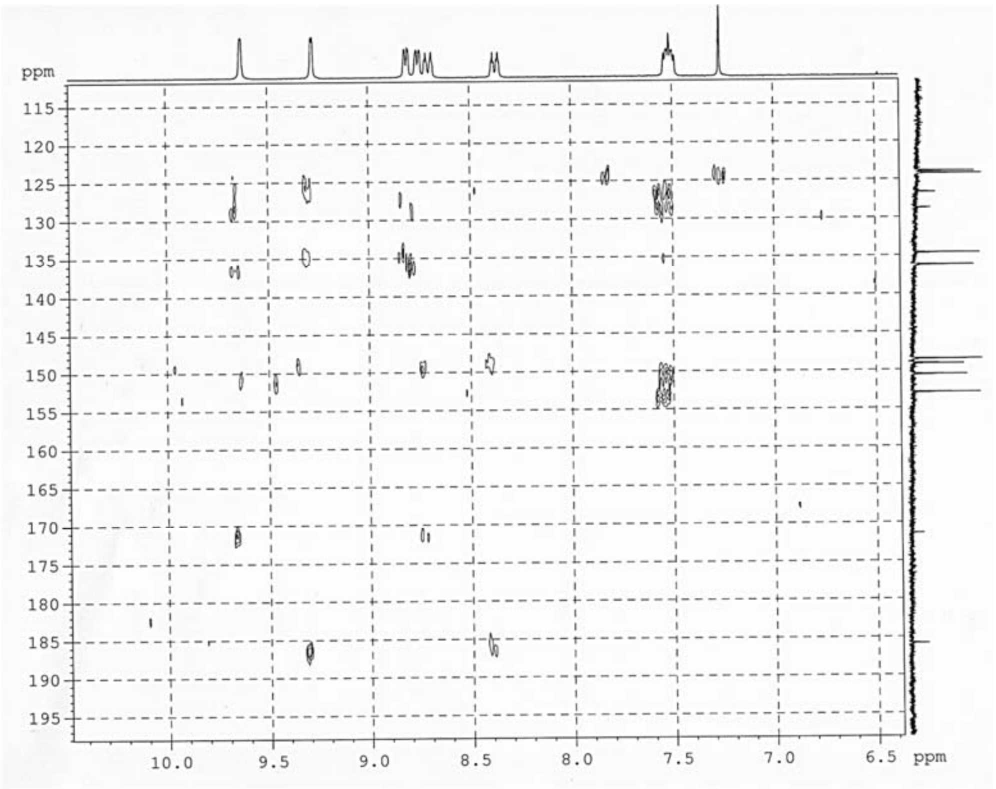

Figure S7. HMBC spectrum of **1** in $CDCl_3$

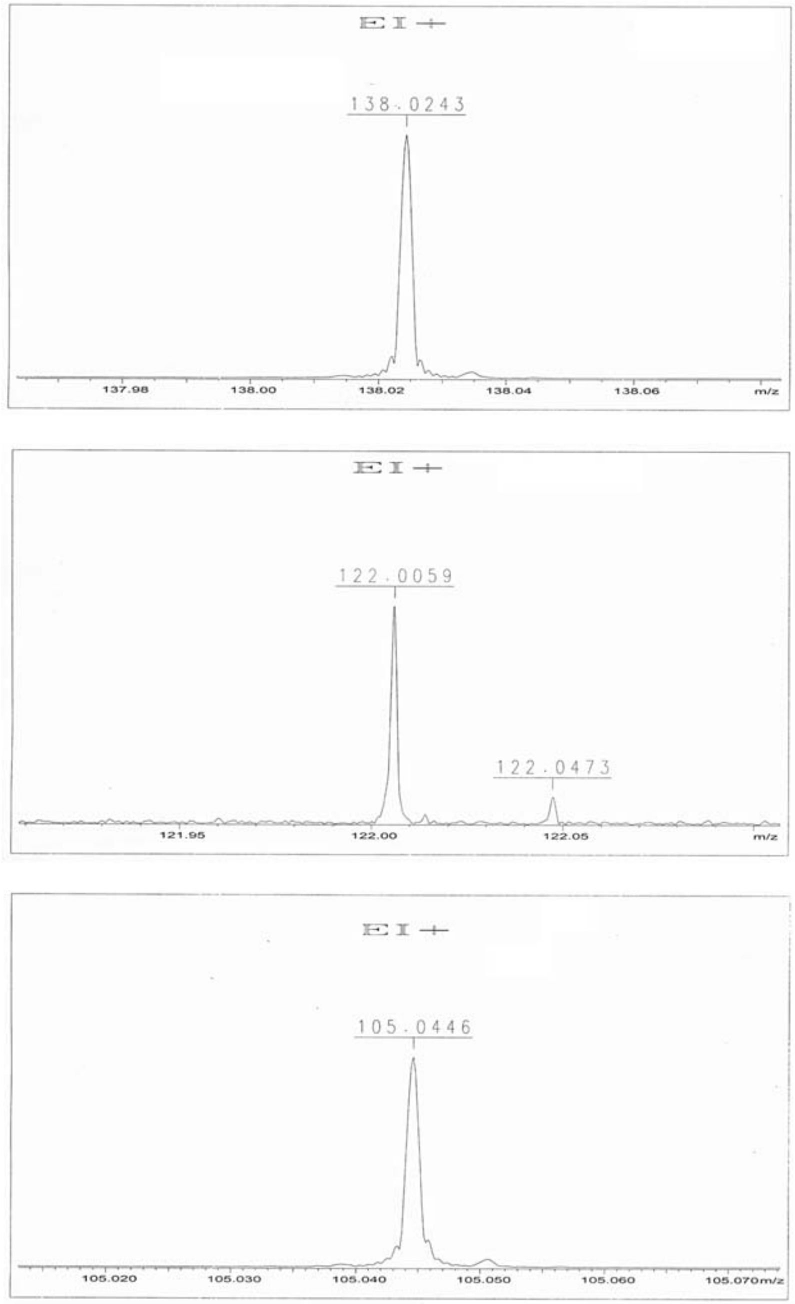

Figure S8. HREIMS spectrum of **2** (M^+ and major ion peaks)

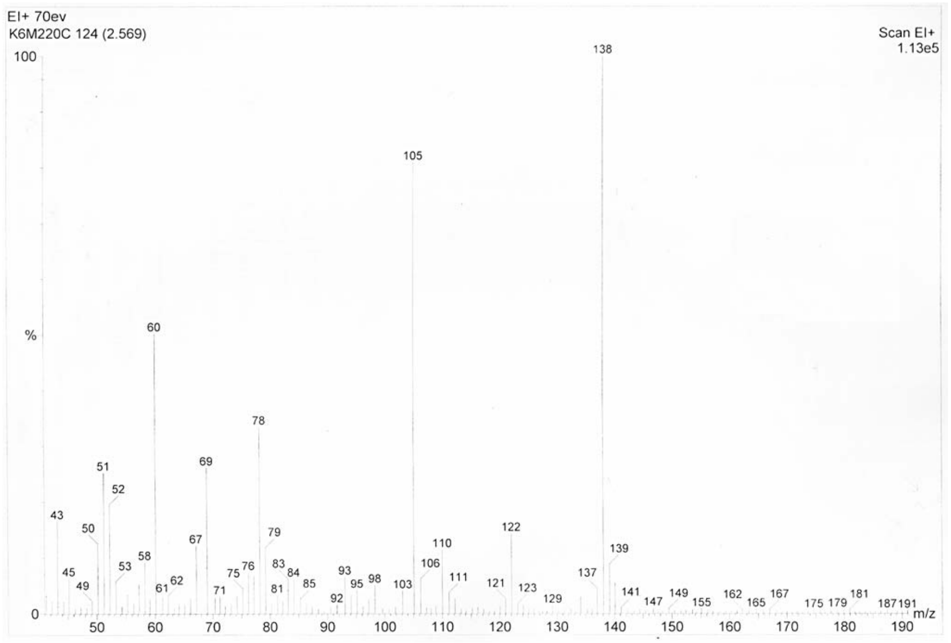

Figure S9. EIMS spectrum of **2**

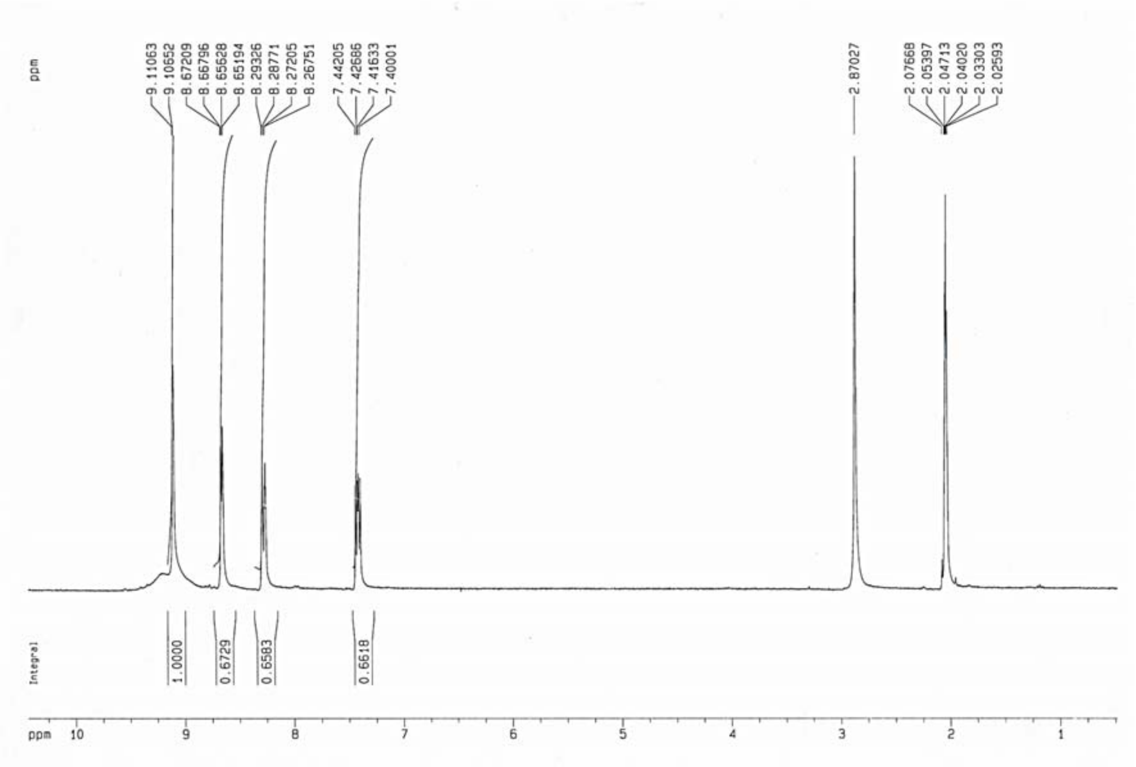

Figure S10. ^{1}H NMR spectrum of **2** in acetone-d_6 at 300 MHz

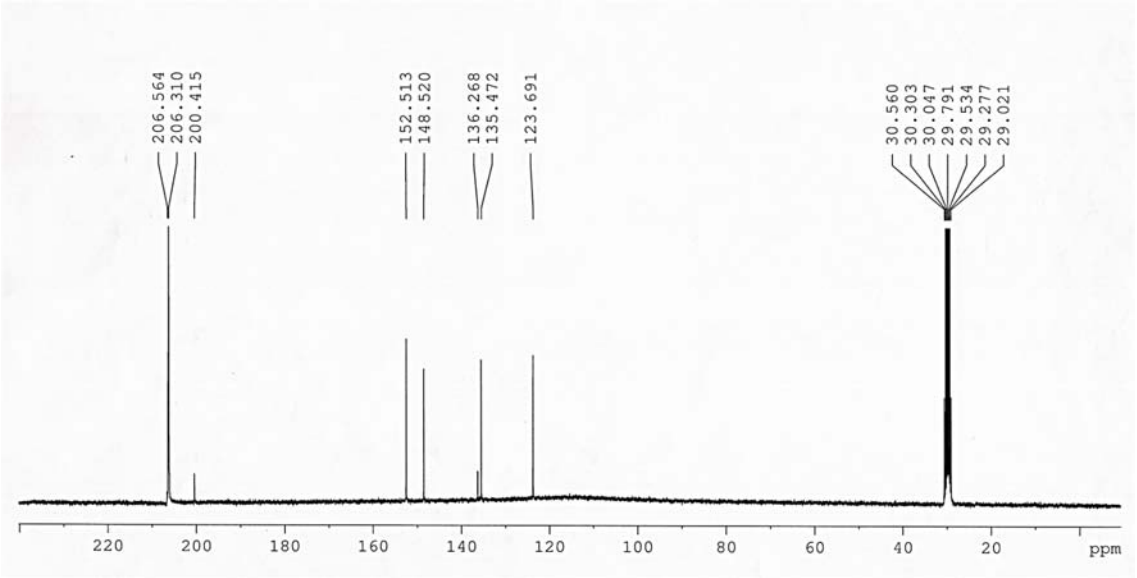

Figure S11. ^{13}C NMR spectrum of **2** in acetone-d_6 at 75 MHz

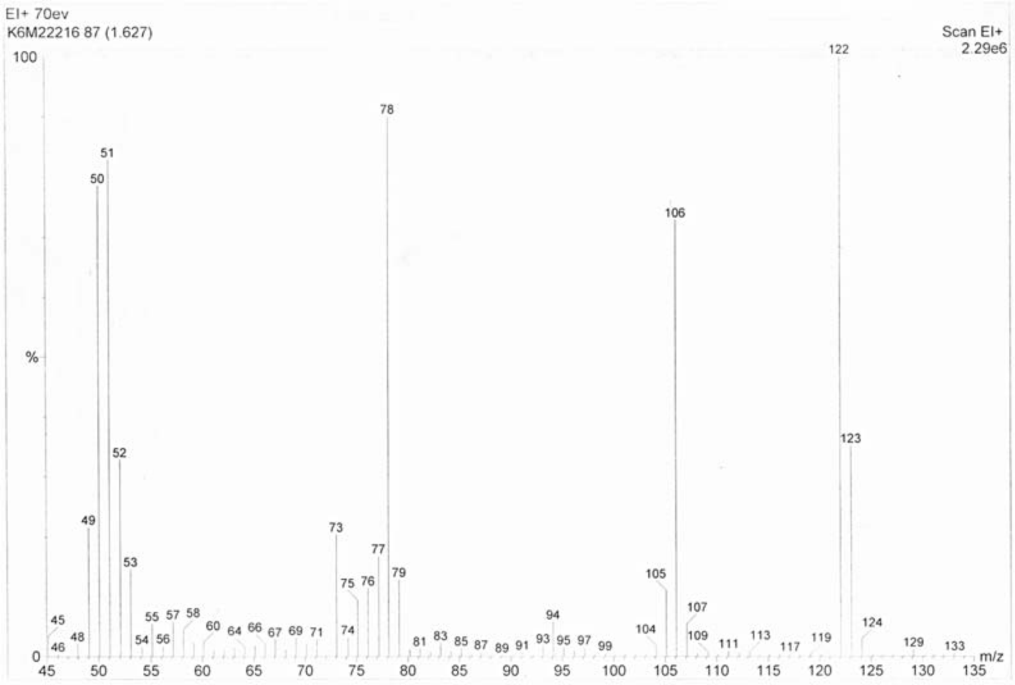

Figure S12. EIMS spectrum of **3**

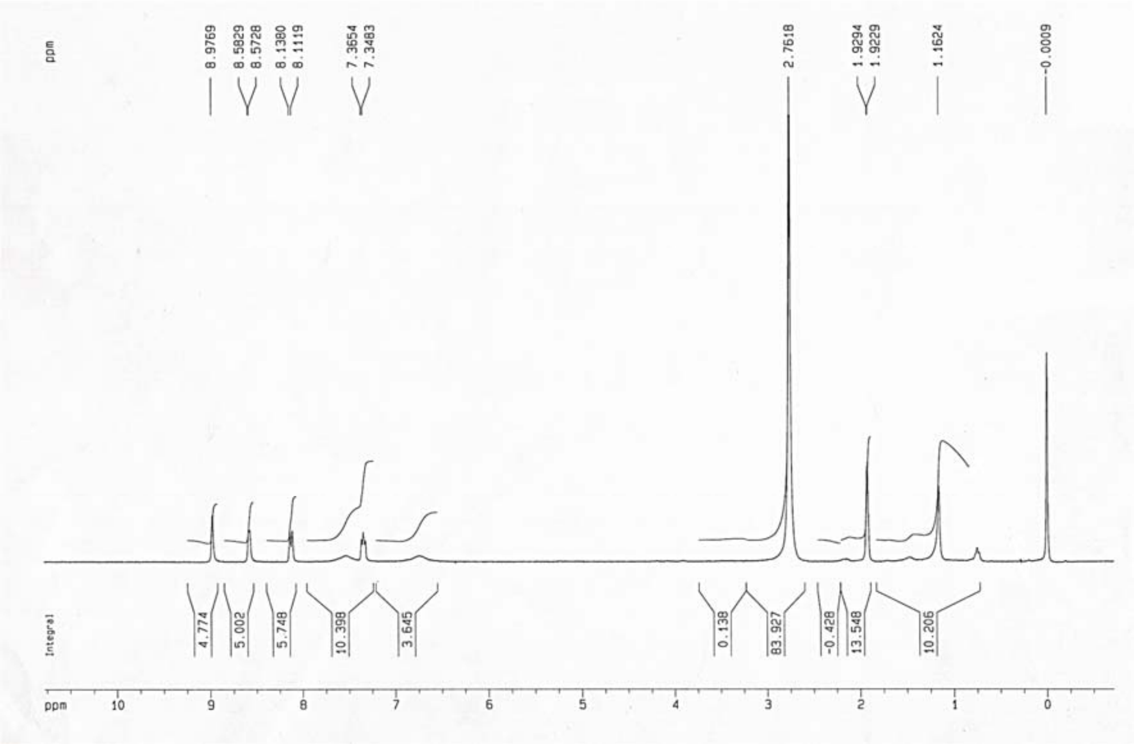

Figure S13. ^{1}H NMR spectrum of **3** in acetone-d_6 at 300 MHz

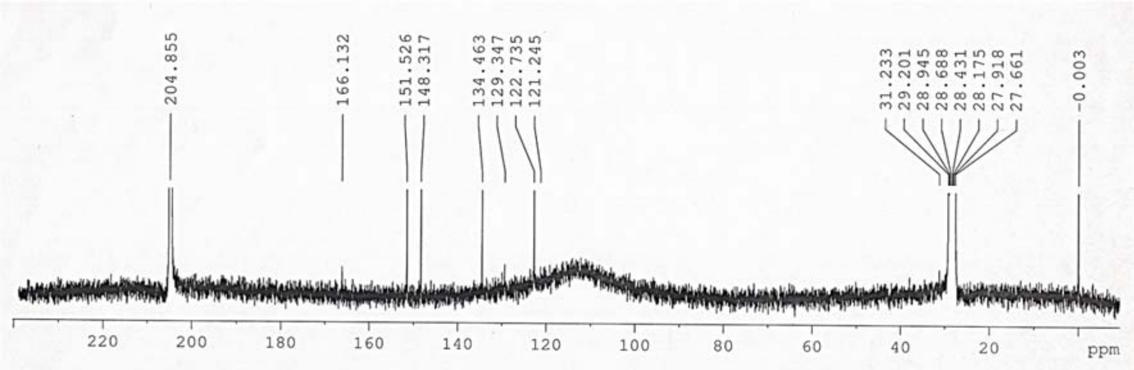

Figure S14. ^{13}C NMR spectrum of **3** in acetone-d_6 at 75 MHz

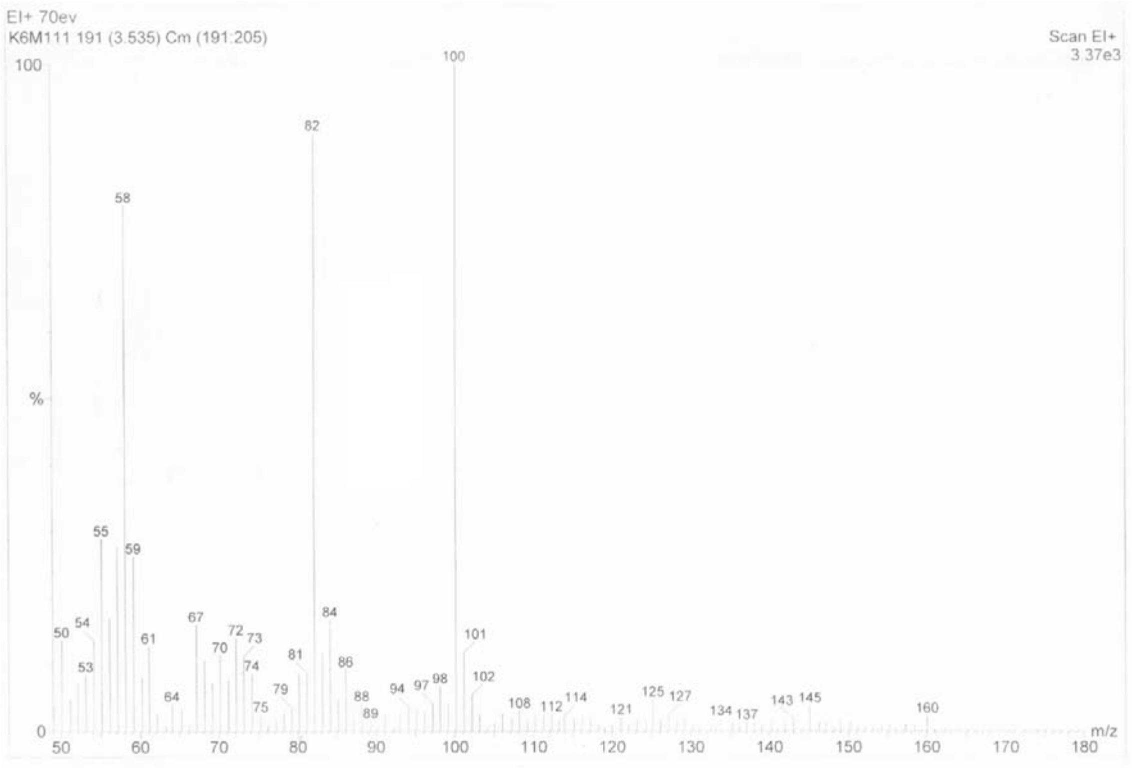

Figure S15. EIMS spectrum of **4**

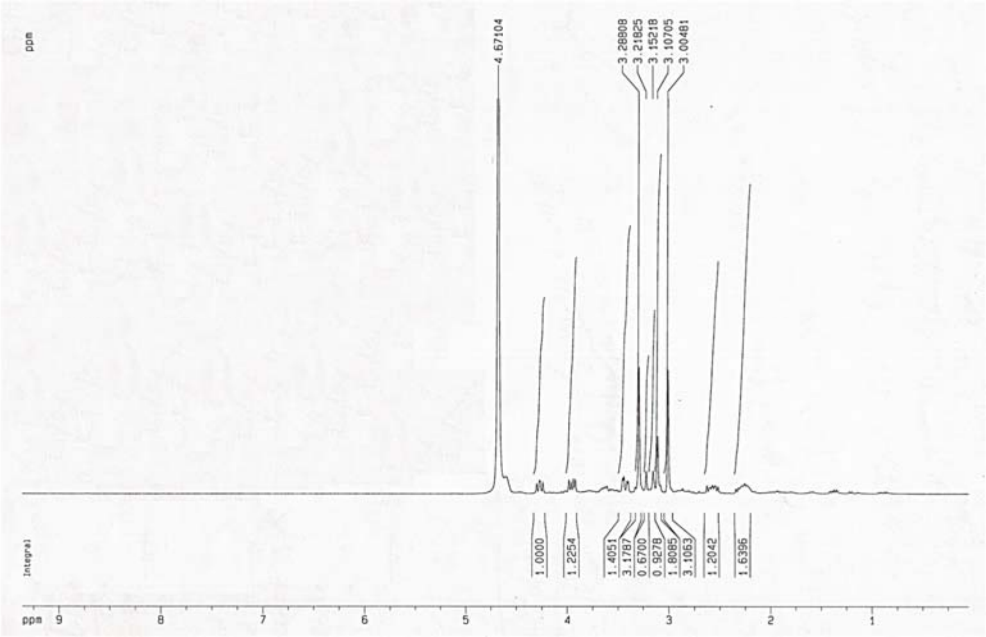

Figure S16. ^{1}H NMR spectrum of **4** in D_2O at 300 MHz

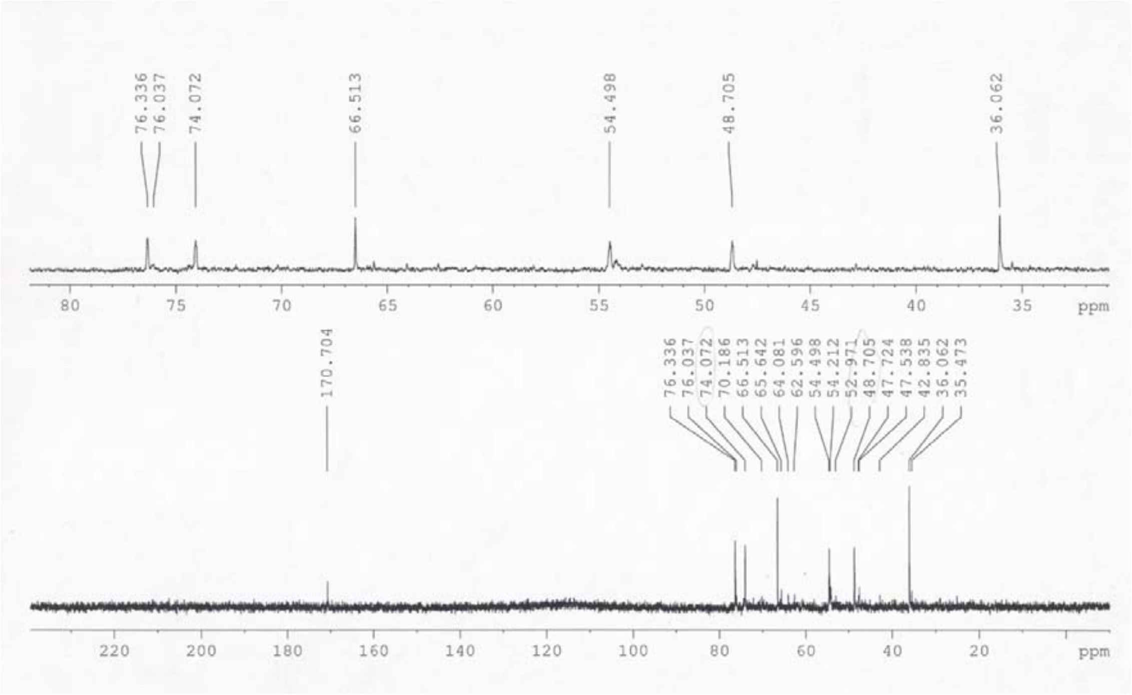

Figure S17. ^{13}C NMR spectrum of **4** in D_2O at 75 MHz

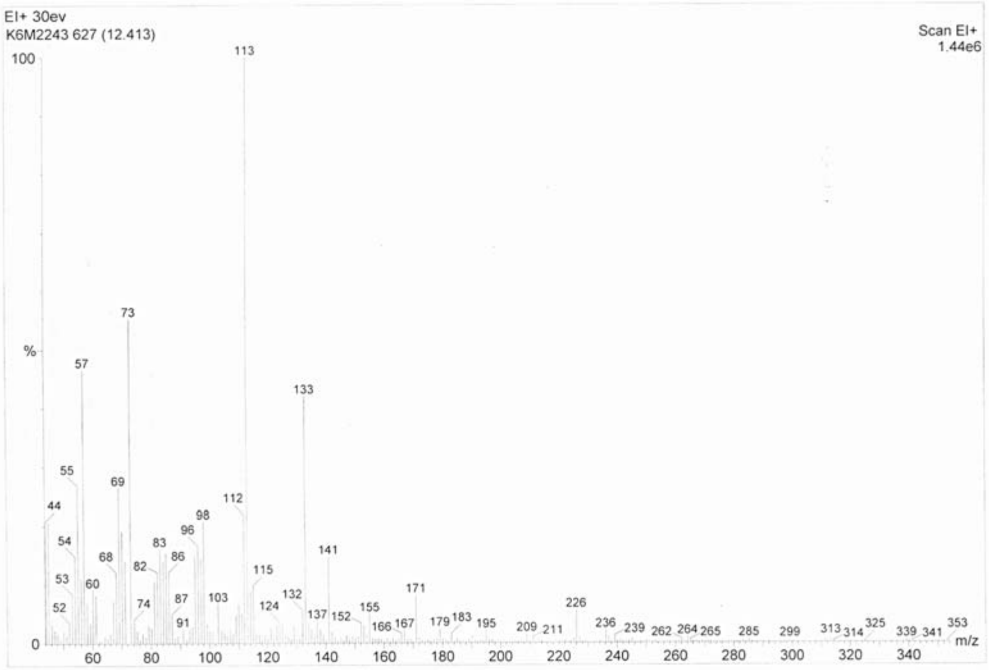

Figure S18. EIMS spectrum of **5**

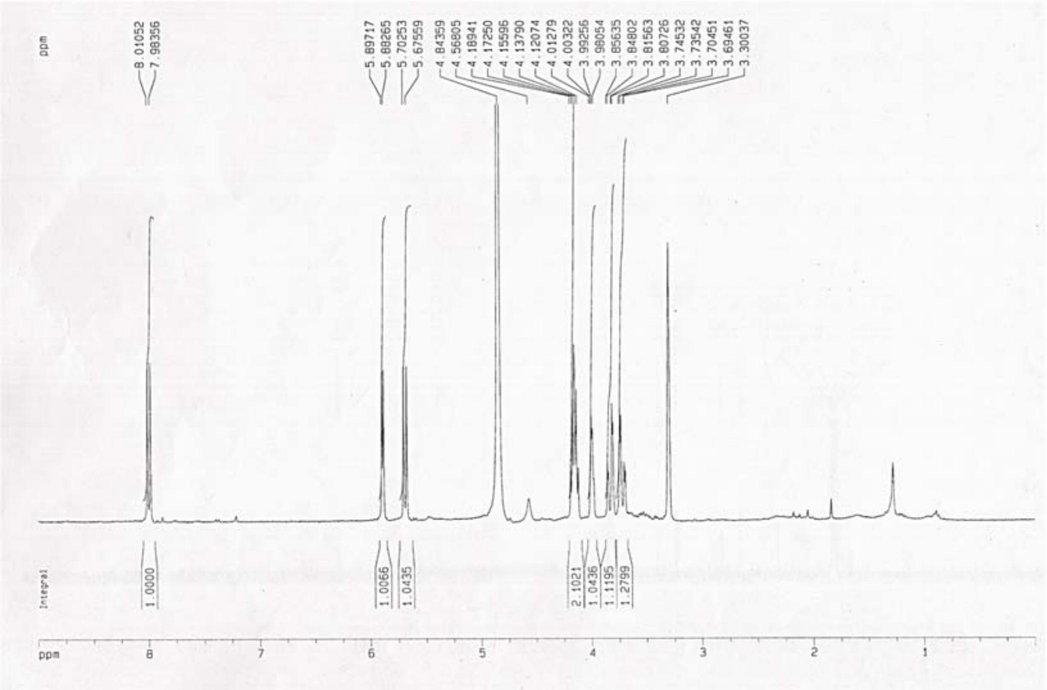

Figure S19. ^{1}H NMR spectrum of **5** in CD_3OD at 300 MHz

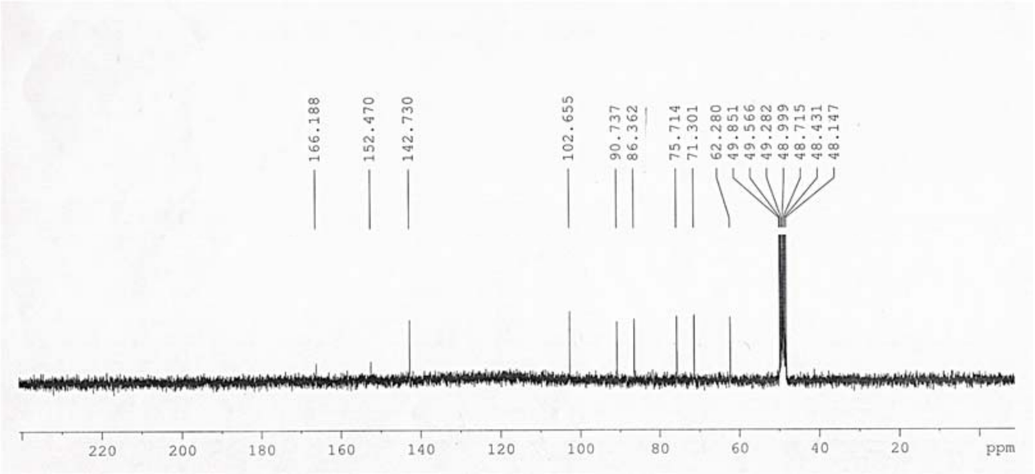

Figure S20. ^{13}C NMR spectrum of **5** in CD_3OD at 75 MHz

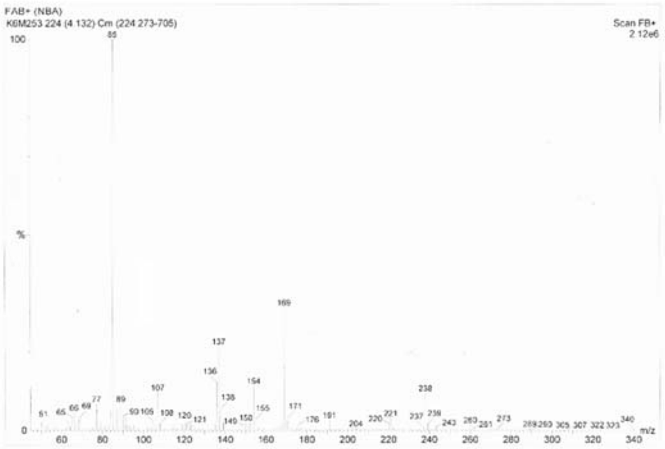

Figure S21. FABMS spectrum of **6**

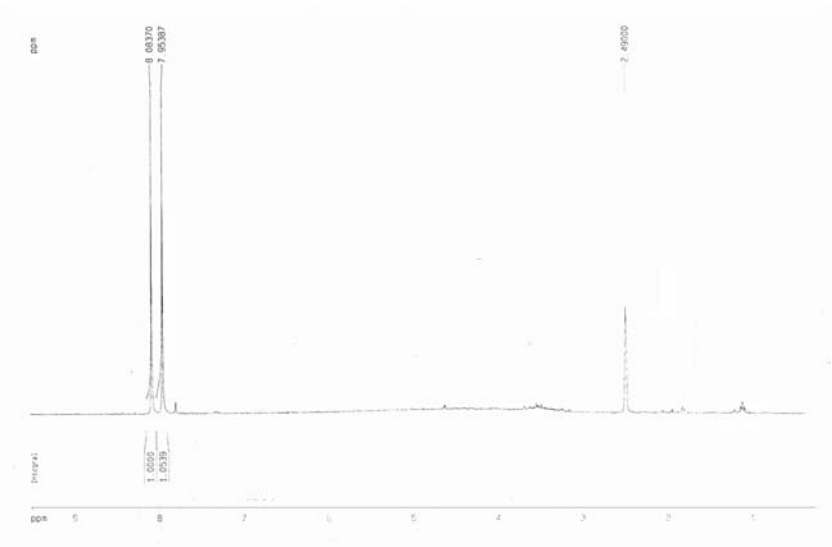

Figure S22. ^{1}H NMR spectrum of **6** in DMSO-d_6 at 300 MHz

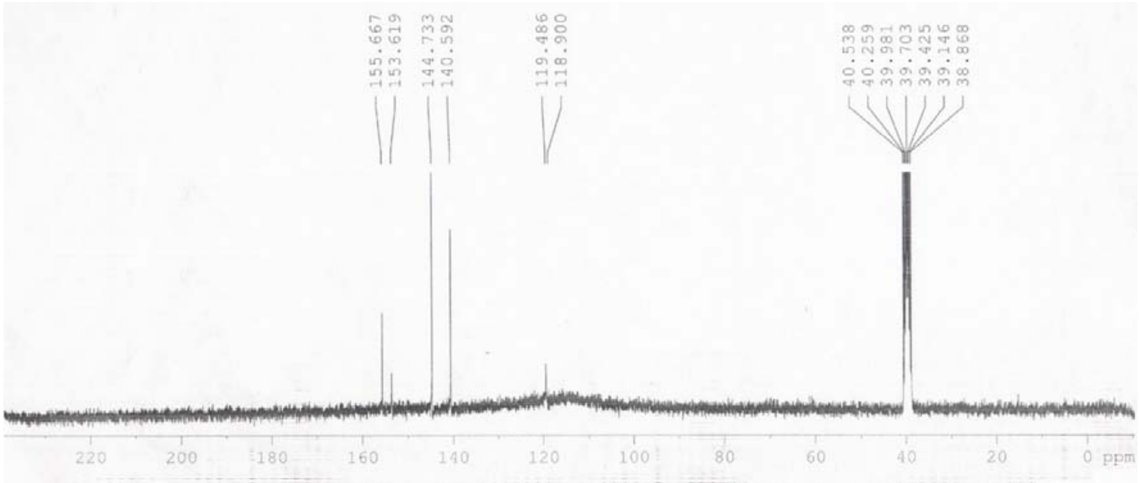

Figure S23. ^{13}C NMR spectrum of **6** in DMSO-d_6 at 75 MHz

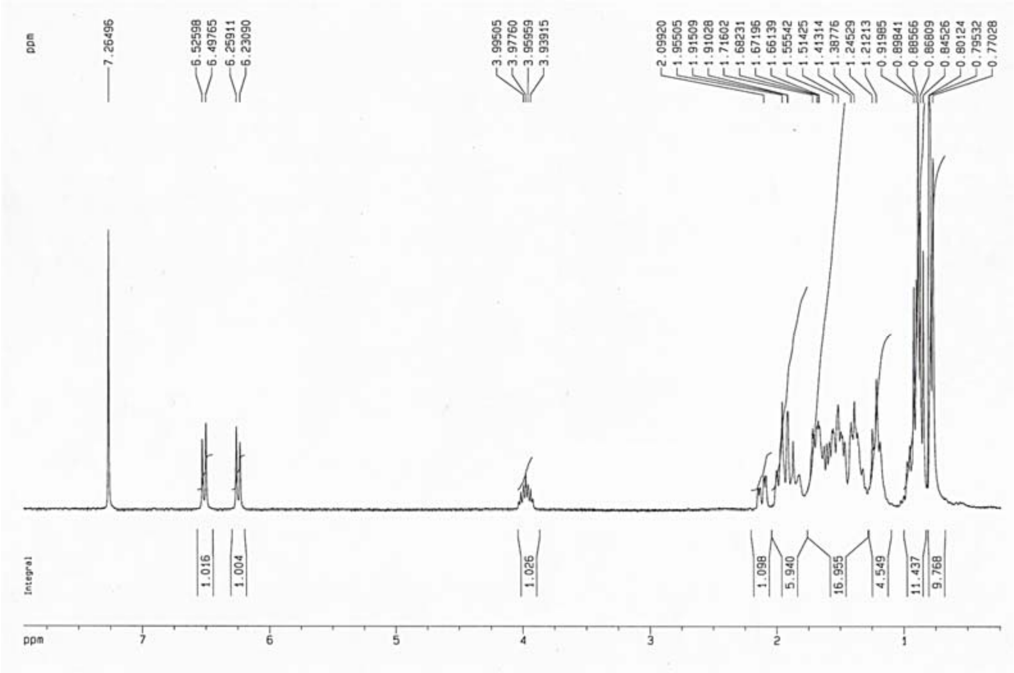

Figure S24. ^{1}H NMR spectrum of **7** in $CDCl_3$ 300 MHz

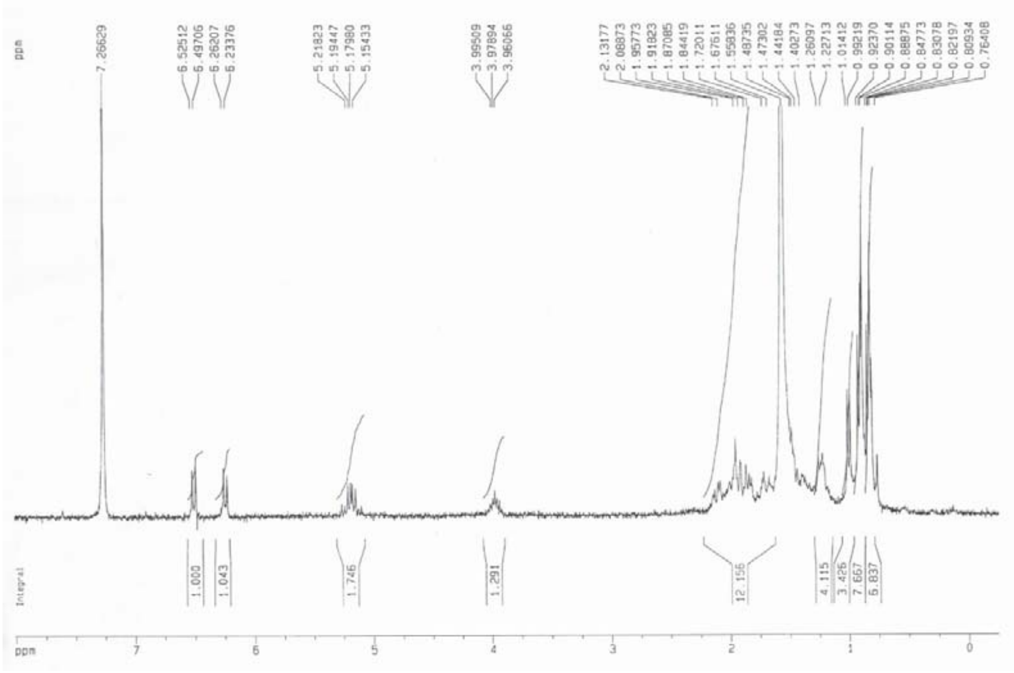

Figure S25. ^{1}H NMR spectrum of **8** in $CDCl_3$ 300 MHz

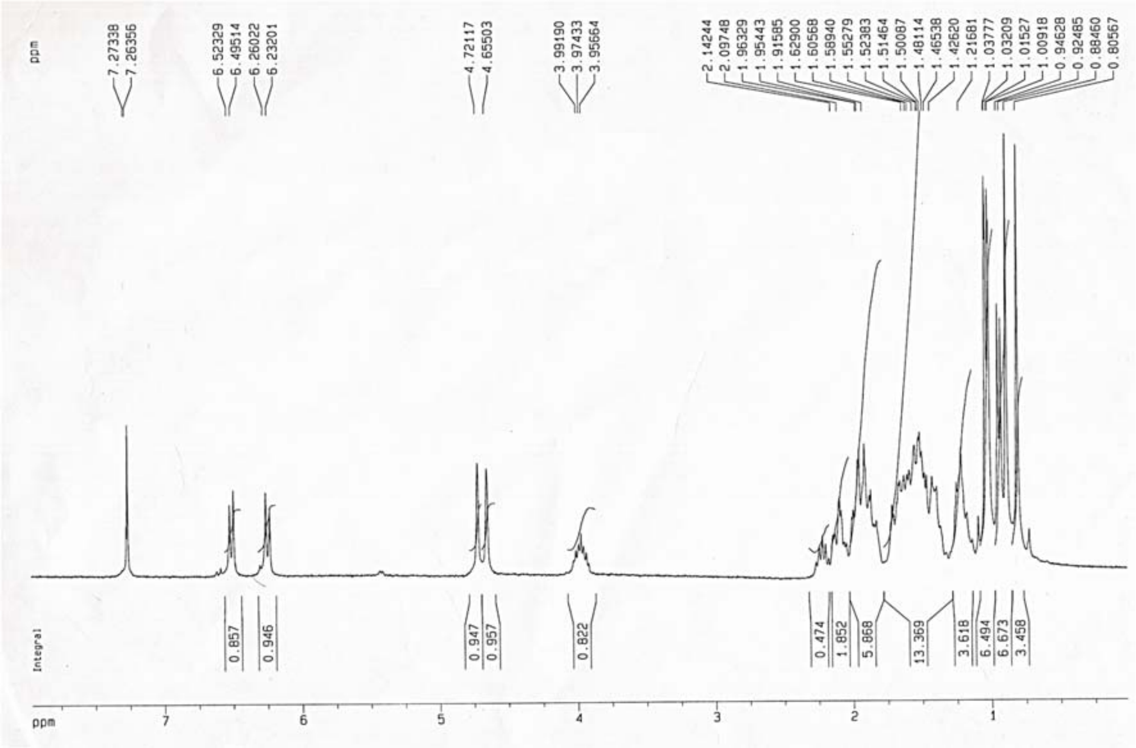

Figure S26. ^{1}H NMR spectrum of **9** in $CDCl_3$ 300 MHz

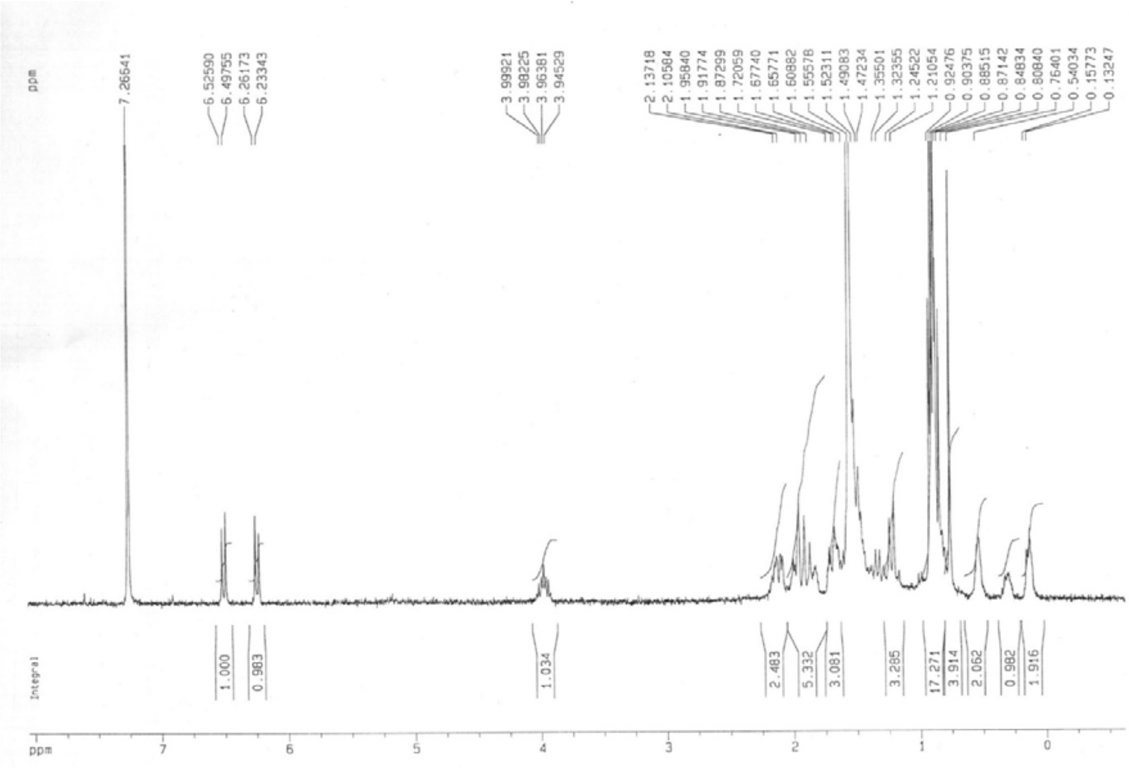

Figure S27. ^{1}H NMR spectrum of **10** in $CDCl_3$ 300 MHz
